# Structure-aware state space modeling with multi-scale feature fusion for railway scene segmentation

**DOI:** 10.1038/s41598-026-47010-x

**Published:** 2026-04-17

**Authors:** Huijin Fu, Zhen Ma, Xue Yang, Wanpeng Zhang, Lei Hu, Ke Jiang

**Affiliations:** 1 China Academy of Railway Sciences, Beijing, 100081 China; 2 Beijing Jingwei Information Technologies Co., Ltd, Beijing, 100081 China

**Keywords:** Network traffic prediction, Wavelet decomposition, State space models, Mamba architecture, Fourier analysis, Multi-scale temporal modeling, Intelligent resource management, Traffic-aware selective mechanisms, Frequency domain processing, Distributed network systems, Long-range dependency modeling, Engineering, Mathematics and computing

## Abstract

Edge-deployed railway safety monitoring demands pixel-perfect semantic segmentation under extreme constraints: detection failures threaten operational safety, yet trackside devices must process diverse environmental conditions (fog, rain, nighttime) at over 30 FPS with under 50M parameters. Traditional CNNs lack long-range modeling capabilities for distant obstacle detection, while Transformer-based methods impose prohibitive quadratic computational complexity unsuitable for edge deployment. We present HybridSeg, a novel architecture that reformulates visual state space modeling as a controllable Markov Decision Process, enabling context-adaptive information propagation through reinforcement learning. Our approach integrates: (1) meta-learned state space dynamics via Proximal Policy Optimization, where learned policies dynamically adjust state transition parameters based on gradient feedback and hidden state statistics, the first application of RL-based parameter adaptation to visual state space models; (2) Structure-Aware Deformable Mamba blocks combining four-directional scanning with deformable spatial attention for irregular geometry handling; (3) cross-scale attention fusion across four pyramid levels with learnable inter-scale dependency modeling; (4) explicit multi-scale consistency constraints stabilizing training and improving generalization. Evaluated on 8,000 railway surveillance images spanning four environmental conditions, HybridSeg achieves 92.34 ± 0.25% mIoU and 97.82 ± 0.12% pixel accuracy at 38.52 FPS with 45.28M parameters–outperforming state-of-the-art CNN, Transformer, and Mamba methods by 1.61-3.16% in accuracy while delivering 2.31× faster inference than comparable approaches. The architecture demonstrates robust cross-domain transfer (89.53% CDR) and competitive performance on Cityscapes (85.73%), CamVid (87.25%), and ADE20K (48.53%), validating practical deployment for safety-critical edge applications.

## Introduction

### Background

Railway safety monitoring systems demand pixel-perfect semantic segmentation under extreme operational constraints: trackside vision systems must detect personnel, foreign objects, and infrastructure anomalies in real-time while operating on resource-limited edge devices without cloud connectivity^1,2^. Unlike conventional computer vision tasks, railway monitoring presents unique challenges where detection failures directly threaten operational safety, requiring robust performance across diverse environmental conditions (fog, rain, nighttime illumination) while maintaining strict computational budgets (less than 50M parameters, over 30 FPS processing speed)^3–8^. As highlighted by Minaee et al.^3^, the convergence of deep learning and edge computing has prompted the development of novel architectures that can leverage sophisticated neural models while maintaining the efficiency required for practical deployment. Modern applications demand precise pixel-level understanding that can effectively operate within hardware constraints, handle diverse imaging conditions, and adapt to varying object scales in challenging environmental scenarios, where traditional computer vision approaches often fail to deliver adequate performance^4,9–12^.

U-Net has emerged as a prominent framework for semantic segmentation due to its modular encoder-decoder design, computational efficiency, and adaptability across diverse imaging modalities. Azad et al.^13^ demonstrated that the U-Net’s skip connection paradigm provides an effective foundation for multi-scale feature processing, addressing scale and complexity challenges in modern vision applications. Recent extensions focus on improving performance through improved backbone architectures, dynamic feature fusion mechanisms, and adaptive attention modules that integrate Transformer-based components for enhanced context modeling^6,14,15^. However, Transformer-based approaches impose quadratic computational complexity (O($\textrm{N}^{2}$)), which is prohibitive for edge deployment–a single 256 × 256 image requires approximately 4GB of memory for self-attention computation, rendering real-time processing infeasible on trackside devices^6,15^.

Recent developments in state space models, particularly Mamba architectures, have introduced promising alternatives for addressing computational efficiency challenges through linear-complexity operators. Li et al.^16^ demonstrated that VideoMamba successfully addresses the dual challenges of local redundancy and global dependencies through linear-complexity operators that enable efficient long-range modeling. Building upon these advances, Ma and Wang^17^ introduced Semi-Mamba-UNet, which integrates visual Mamba architectures with conventional CNN frameworks to process long-range dependencies while requiring substantially reduced computational resources. Their approach demonstrates superior performance compared to traditional CNN- or ViT-based methods while maintaining efficiency suitable for edge deployment. However, existing Mamba-based methods face fundamental limitations: (1) fixed scanning patterns fail to capture complex 2D spatial structures (e.g., curved tracks, irregular object geometries), (2) static state transition matrices cannot adapt to varying visual contexts (high-frequency textures vs. homogeneous regions), and (3) single-scale processing struggles with objects spanning vastly different scales (10-1000 pixels for distant vs. near-field obstacles). Contemporary advances have also explored boundary-aware processing^5^, multi-scale context modeling^10^, and attention mechanisms for enhanced visual analysis^6,12,18–20^, collectively advancing efficient segmentation solutions for resource-constrained applications.

### Motivation and contributions

Despite significant progress in real-time semantic segmentation systems, existing approaches fail to simultaneously address three critical requirements for safety-critical edge deployment: (1) linear computational complexity for real-time processing, (2) adaptive information propagation based on visual context, and (3) robust multi-scale feature integration across diverse environmental conditions. Current methods predominantly focus on either localized processing via lightweight CNNs or global context modeling via computationally intensive Transformer-based architectures, without effectively integrating efficient processing capabilities that can preserve multi-scale feature coordination while maintaining the computational efficiency required for edge deployment. The emergence of state space models presents unique opportunities for linear-complexity processing mechanisms; however, their application to efficient visual segmentation remains limited by rigid architectural designs that cannot dynamically adjust to varying scene characteristics.

To address these fundamental limitations, this work proposes HybridSeg, a comprehensive multi-scale vision architecture that reformulates visual state space modeling as a controllable Markov Decision Process. Our approach systematically integrates four synergistic architectural innovations to achieve robust segmentation performance while maintaining the efficiency and adaptability required for practical edge deployment scenarios. Specifically, we make the following contributions:

- We design a novel efficient architecture that integrates Structure-Aware Preprocessing (SAP) and Structure-Aware Deformable Mamba (SADM) blocks, enabling adaptive visual processing through multi-directional scanning patterns (horizontal, vertical, diagonal, anti-diagonal) and deformable spatial attention mechanisms that capture long-range dependencies and complex structural patterns while adapting to irregular object geometries through learned offset prediction and attention modulation strategies.
- We propose the first application of reinforcement learning-based meta-learning to visual state space models, formulating state transition parameter adaptation as a Markov Decision Process solved via Proximal Policy Optimization (PPO). Our learned policy network dynamically adjusts state space matrices based on current hidden states, gradient feedback from downstream segmentation loss, and statistical context (mean, variance, inter-directional correlation), enabling context-dependent information propagation that adapts to varying scene characteristics–e.g., increasing state persistence in homogeneous regions while enhancing input responsiveness near object boundaries.
- We introduce a Multi-Scale Feature Fusion (MSFF) framework with cross-scale attention mechanisms that enables progressive coordination between four hierarchical pyramid levels and implements inter-scale dependency modeling through learnable fusion weights, effectively handling objects of varying sizes and complex boundaries while enhancing both local detail preservation and global context representation through comprehensive feature alignment and attention-based fusion strategies.
- We present Cross-Scale Consistency (CSC) constraints that enforce feature and prediction consistency across different scales through explicit loss formulations, ensuring training stability and improving generalization while maintaining structural coherence throughout the processing pipeline. We provide theoretical analysis establishing Lipschitz continuity bounds and convergence guarantees for the complete architecture.
- We conduct a comprehensive experimental evaluation on four benchmark datasets, demonstrating that HybridSeg achieves 92.34% mIoU on railway surveillance (outperforming state-of-the-art CNN-based, Transformer-based, and existing Mamba-based approaches by 1.61-3.16% in accuracy) while maintaining 38.52 FPS at 45.28M parameters –2.31× faster inference than comparable methods. The architecture exhibits robust cross-domain performance (89.53% CDR) and competitive results on Cityscapes (85.73%), CamVid (87.25%), and ADE20K (48.53%), validating practical deployment for safety-critical edge computing platforms.

The remainder of this paper is organized as follows. Section Related work reviews the existing literature on efficient visual processing, state space models, and multi-scale coordination approaches. Section Method describes the architecture and technical innovations of our HybridSeg framework. Section Experiment presents the experimental setup and evaluation methodology. Section Results analyzes the performance evaluation results and comparative studies. Section Conclusion concludes the paper and discusses future research directions for efficient edge-deployable segmentation systems. (Table 1)

**Table 1:** Contrasting our work to existing image segmentation studies.

Feature	Ref										
	^21^	^22^	^23^	^24^	^25^	^26^	^17^	^14^	^10^	^6^	Proposed work
Mamba/state space models				$\checkmark$	$\checkmark$	$\checkmark$	$\checkmark$				$\checkmark$
Structure-aware processing											$\checkmark$
Deformable attention			$\checkmark$								$\checkmark$
Multi-scale feature fusion	$\checkmark$		$\checkmark$					$\checkmark$	$\checkmark$	$\checkmark$	$\checkmark$
Cross-scale consistency											$\checkmark$
Linear complexity modeling				$\checkmark$	$\checkmark$	$\checkmark$	$\checkmark$				$\checkmark$

## Related work

### Encoder-decoder architectures for semantic segmentation

Encoder-decoder architectures have evolved significantly for semantic segmentation tasks, with recent advances integrating hierarchical feature processing and multi-scale coordination strategies to address complex visual perception challenges in resource-constrained environments^3,21–23,27–31^. Zhou et al.^21^ proposed UNet++, addressing critical limitations of conventional architectures by redesigning skip connections to aggregate features of varying semantic scales across encoder-decoder hierarchies. Their approach introduces nested dense skip pathways that enable progressive feature refinement, demonstrating consistent improvements across multiple medical imaging scenarios. To further enhance long-range dependency modeling, Chen et al.^22^ developed TransAttUnet, incorporating transformer-based self-attention modules that combine local convolutional features with global contextual information. Their framework employs multi-scale feature aggregation to strengthen semantic representation while preserving fine-grained spatial details across hierarchical layers.

In the context of multi-modal visual perception, Zhang et al.^23^ introduced CMX, a unified cross-modal fusion framework for RGB-X semantic segmentation that generalizes across diverse sensor modalities, including depth, thermal, polarization, event, and LiDAR data. Their approach deploys a Cross-Modal Feature Rectification Module to calibrate complementary features through cross-modality information exchange, followed by a Feature Fusion Module for comprehensive context integration, achieving state-of-the-art performance across multiple RGB-X benchmarks. Early foundational works such as SegNet^27^ established encoder-decoder paradigms with efficient memory management, while Feature Pyramid Networks^29^ introduced multi-scale processing concepts through hierarchical feature pyramids, and recent transformer-based approaches like META-Unet^28^ have explored efficient attention mechanisms for medical image segmentation, as comprehensively surveyed by^3^. However, these methods face fundamental trade-offs: CNN-based approaches lack sufficient long-range modeling capabilities for distant object detection, while Transformer-based architectures impose quadratic computational complexity that is prohibitive for edge deployment scenarios.

### State space models and mamba architectures for vision

State space models and Mamba architectures have emerged as promising alternatives offering linear computational complexity while maintaining strong sequence modeling capabilities, with recent extensions to visual tasks demonstrating significant potential across medical image analysis, low-level vision restoration, and general scene understanding^19,24–26,32–37^. Liu et al.^24^ introduced Swin-UMamba, a Mamba-based U-Net variant that leverages state space models’ linear complexity for efficient medical image segmentation. Their approach addresses the computational barriers posed by transformer-based models in resource-constrained scenarios, where their quadratic complexity creates deployment challenges. By integrating Swin Transformer blocks with Mamba layers through self-supervised pre-training, they demonstrate superior performance over seven state-of-the-art CNN-based, transformer-based, and existing Mamba-based methods across diverse medical imaging modalities.

Addressing fundamental limitations in Vision Mamba architectures, Wang et al.^25^ identified and mitigated high-norm token artifacts in feature processing, specifically problematic patterns that emerge in low-information regions and are more severe in Vision Mamba than in Vision Transformers. Their MambaReg approach introduces register tokens with two key modifications: evenly distributing regularization signals across the architecture and recycling information across layers to improve feature stability. This solution produces cleaner feature maps focused on semantically meaningful regions, achieving enhanced efficiency while successfully scaling to large model configurations. In the context of low-level vision tasks, Shi et al.^26^ proposed VmambaIR, introducing state-space models into comprehensive image restoration (denoising, deblurring, deraining) via omni-selective scanning mechanisms. Their approach overcomes unidirectional processing limitations by efficiently modeling information flows across all spatial directions (horizontal, vertical, diagonal, anti-diagonal), achieving state-of-the-art restoration performance with significantly reduced computational requirements. Recent developments have also explored interpretability aspects^32^, graph-based applications^33^, selective visual prompting^34^, and frequency-enhanced lightweight architectures^35,38^, collectively advancing the state space model paradigm for visual understanding.

Despite these advances, existing Mamba-based methods face critical limitations for safety-critical edge deployment: (1) fixed unidirectional or bidirectional scanning patterns fail to capture complex 2D spatial structures and irregular object geometries; (2) static state transition parameters cannot adapt to varying visual contexts–e.g., homogeneous background regions vs. high-frequency texture near object boundaries; (3) lack of explicit multi-scale feature integration mechanisms to handle objects spanning vastly different scales (10-1000 pixels). Our work addresses these fundamental challenges through adaptive state space control via reinforcement learning and comprehensive multi-scale consistency constraints.

### Multi-scale feature fusion and hierarchical processing

Multi-scale feature fusion and hierarchical processing have become fundamental components in modern semantic segmentation systems, with significant advances in attention-guided fusion mechanisms, pyramid architectures, and geometric structure understanding for enhanced visual representation learning^39–48^. Van Quyen and Kim^39^ addressed critical limitations in Feature Pyramid Networks, where naive fusion methods combine predictions across different scales without accounting for their complementary characteristics. Their approach introduces dual prediction fusion mechanisms that leverage scale-specific strengths: low-resolution features provide superior semantic understanding of large objects, while high-resolution features capture fine-grained details of small targets. The framework employs attention-based multi-scale fusion, identifying unreliable predictions at each scale and compensating them with information from complementary scales, achieving 77.9% mIoU at 62 FPS for real-time semantic segmentation and 44.1% mIoU on complex panoptic segmentation tasks.

From a theoretical perspective, Mitra et al.^42^ established comprehensive foundations for structure-aware shape processing, emphasizing analysis beyond local geometry to understand global structural patterns and spatial arrangements. Their framework consists of two key phases: an analysis phase that extracts structural relationships via symmetry detection and part decomposition, and a synthesis phase that leverages these relationships for shape exploration, editing, and generation. This structure-aware paradigm focuses on high-level geometric arrangements and inter-part relations rather than low-level surface details, providing essential theoretical groundwork for linking geometric structure to functional semantics and enabling intelligent shape manipulation algorithms. Building upon these principles, Zhu et al.^46^ proposed a multi-resolution hybrid CNN-Transformer architecture for medical image segmentation. Their approach features attention-guided multi-scale fusion with dual-path processing in encoders, an advanced feature aggregation module in decoders that models inter-dependencies across hierarchical levels through cross-attention mechanisms, and multi-scale feature activation with multi-layer perceptron blocks for high-level semantic learning. The implementation of progressive feature refinement enriches hierarchical representations and ensures fine-grained outputs across different scales, demonstrating superior performance on diverse medical imaging modalities including CT, MRI, and ultrasound. Recent complementary advances include augmented feature pyramid networks^40^, structure-aware generative modeling^43^, cross-scale fusion transformers^44^, and semantic alignment using global-local features^47^, collectively advancing multi-scale processing capabilities for visual understanding.

However, existing multi-scale fusion methods lack explicit mechanisms to enforce consistency across hierarchical levels during training, leading to potential scale-specific overfitting and degraded generalization to unseen environmental conditions. Our Cross-Scale Consistency (CSC) training addresses this limitation through explicit feature alignment and prediction consistency constraints, ensuring robust performance across diverse operational scenarios critical for safety-critical applications.

## Method

In this section, we introduce our improved hybrid network architecture for distributed multi-modal visual perception, called HybridSeg. Our method tackles key challenges in network-based visual processing by combining structure-aware Mamba modeling, deformable attention mechanisms, and multi-scale feature coordination within a self-organizing UNet-based architecture. We systematically incorporate four main architectural innovations to achieve reliable distributed visual perception results while maintaining computational efficiency suitable for resource-constrained IoT environments.

### Overall architecture

**Fig. 1:**
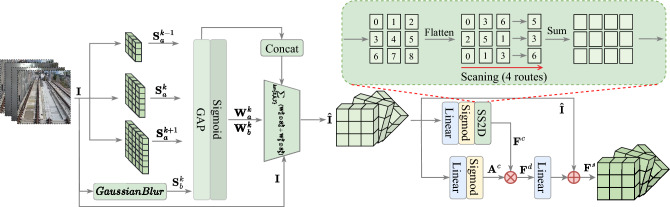
HybridSeg framework overview.

Figure 1 illustrates our proposed HybridSeg network framework. Built on the classic UNet encoder-decoder architecture with network-adaptive enhancements, our distributed processing network features four main innovations: (1) Structure-Aware Preprocessing (SAP) for distributed input enhancement, (2) Structure-Aware Deformable Mamba (SADM) blocks replacing traditional convolutional layers to enable efficient inter-node coordination, (3) Multi-Scale Feature Fusion (MSFF) with cross-scale attention for hierarchical network coordination, and (4) Cross-Scale Consistency (CSC) to improve training stability across distributed processing nodes.

Given input image $\textbf{I} \in \mathbb {R}^{C \times H \times W}$ from distributed sensors, our network framework generates segmentation mask $\textbf{M} \in \mathbb {R}^{1 \times H \times W}$ through:

1 $$\begin{aligned} \hat{\textbf{I}}&= \text {SAP}(\textbf{I}) \in \mathbb {R}^{C \times H \times W}, \end{aligned}$$

2 $$\begin{aligned} \{\textbf{F}^1_i\}_{i=1}^{4}&= \text {Encoder}(\hat{\textbf{I}}) \in \mathbb {R}^{C_i \times H_i \times W_i}, \end{aligned}$$

3 $$\begin{aligned} \{\textbf{F}^2_i\}_{i=1}^{4}&= \text {MSFF}(\{\textbf{F}^1_i\}_{i=1}^{4}) \in \mathbb {R}^{C'_i \times H_i \times W_i}, \end{aligned}$$

4 $$\begin{aligned} \textbf{M}&= \text {Decoder}(\{\textbf{F}^2_i\}_{i=1}^{4}) \in \mathbb {R}^{1 \times H \times W}, \end{aligned}$$

5 $$\begin{aligned} \mathscr {L}_c&= \text {CSC}(\{\textbf{F}^1_i\}_{i=1}^{4}, \textbf{M}). \end{aligned}$$

where $\textbf{I}$ denotes the input RGB image with *C* channels, height *H*, and width *W*. $\hat{\textbf{I}}$ represents the network-preprocessed image. $\{\textbf{F}^1_i\}_{i=1}^{4}$ are encoder features at four different network scales with channels $C_i$ and spatial dimensions $H_i \times W_i$. $\{\textbf{F}^2_i\}_{i=1}^{4}$ denote the coordinated multi-scale features with enhanced channels $C'_i$. $\textbf{M}$ is the final binary segmentation mask, and $\mathscr {L}_c$ represents the network consistency loss.

### Structure-aware preprocessing

To enhance structural information preservation for distributed processing environments, we design SAP to extract multi-scale structural features before feeding into the network encoder. This preprocessing addresses the challenge of diverse structural patterns in distributed visual perception tasks while maintaining computational efficiency for IoT deployment.

#### Multi-scale structure extraction

We extract structural features at multiple network scales using different kernel sizes:

6 $$\begin{aligned} \textbf{S}^k_a&= \text {Conv}_{k \times k}(\textbf{I}) \in \mathbb {R}^{C \times H \times W}, \end{aligned}$$

7 $$\begin{aligned} \textbf{S}^k_b&= \textbf{I} - \text {GaussianBlur}(\textbf{I}, \sigma =k) \in \mathbb {R}^{C \times H \times W} \end{aligned}$$

where $k \in \{3, 5, 7\}$ represents different kernel sizes for multi-scale network analysis. $\textbf{S}^k_a$ captures global structural patterns through convolution operations with kernel size $k \times k$. $\textbf{S}^k_b$ preserves local structural details by subtracting Gaussian-blurred features with standard deviation $\sigma =k$ from the original input. *C*, *H*, and *W* denote channel dimension, height, and width respectively.

#### Adaptive feature weighting

Adaptive weights are computed for each network scale to balance different structural components:

8 $$\begin{aligned} \textbf{W}^k_a&= \text {Sigmoid}(\text {Conv}_{1 \times 1}(\text {GAP}(\textbf{S}^k_a))) \in \mathbb {R}^{C \times 1 \times 1}, \end{aligned}$$

9 $$\begin{aligned} \textbf{W}^k_b&= \text {Sigmoid}(\text {Conv}_{1 \times 1}(\text {GAP}(\textbf{S}^k_b))) \in \mathbb {R}^{C \times 1 \times 1} \end{aligned}$$

where $\textbf{W}^k_a$ and $\textbf{W}^k_b$ represent adaptive weights for global and local structural features at scale *k*. $\text {GAP}(\cdot )$ denotes global average pooling operation that reduces spatial dimensions to $1 \times 1$. $\text {Conv}_{1 \times 1}(\cdot )$ applies pointwise convolution, and $\text {Sigmoid}(\cdot )$ ensures weights are in range [0, 1].

The enhanced features are fused through weighted combination:

10 $$\begin{aligned} \textbf{F} = \sum _{k \in \{3,5,7\}} (\textbf{W}^k_a \odot \textbf{S}^k_a + \textbf{W}^k_b \odot \textbf{S}^k_b) \in \mathbb {R}^{C \times H \times W} \end{aligned}$$

where $\textbf{F}^a$ represents the fused structural features. $\odot$ denotes element-wise multiplication. The summation aggregates structural information across all network scales $k \in \{3,5,7\}$.

The final SAP output combines original and enhanced features:

11 $$\begin{aligned} \hat{\textbf{I}} = \textbf{I} + \alpha \textbf{F} \in \mathbb {R}^{C \times H \times W} \end{aligned}$$

where $\hat{\textbf{I}}$ is the preprocessed output image. $\alpha$ is a learnable parameter controlling the enhancement strength. $\textbf{I}$ represents the original input, and $\textbf{F}^a$ denotes the extracted structural features.

### Structure-aware deformable mamba block

The SADM block forms the core of our distributed encoder, replacing traditional convolutional layers to capture long-range dependencies and complex structural patterns suitable for network coordination. This design bridges the gap between local feature extraction and global context modeling while maintaining linear complexity essential for IoT deployment scenarios.

#### Multi-directional scanning

Following structure-aware scanning principles for distributed processing, we implement four scanning directions to capture comprehensive spatial relationships:

12 $$\begin{aligned} \textbf{S}_h&= \text {HorizontalScan}(\textbf{F}) \in \mathbb {R}^{C \times H \times W}, \end{aligned}$$

13 $$\begin{aligned} \textbf{S}_v&= \text {VerticalScan}(\textbf{F}) \in \mathbb {R}^{C \times H \times W}, \end{aligned}$$

14 $$\begin{aligned} \textbf{S}_d&= \text {DiagonalScan}(\textbf{F}) \in \mathbb {R}^{C \times H \times W}, \end{aligned}$$

15 $$\begin{aligned} \textbf{S}_a&= \text {AntiDiagonalScan}(\textbf{F}) \in \mathbb {R}^{C \times H \times W} \end{aligned}$$

where $\textbf{F} \in \mathbb {R}^{C \times H \times W}$ is the input feature map. $\textbf{S}_h, \textbf{S}_v, \textbf{S}_d, \textbf{S}_a$ represent scanned features along horizontal, vertical, diagonal, and anti-diagonal directions respectively. Each scanning operation processes the feature map sequentially along its respective direction to capture directional patterns for network coordination.

For each direction $j \in \{h, v, d, a\}$, the Visual State Space operation is formulated as:

16 $$\begin{aligned} \textbf{P}_j&= e^{\Delta \textbf{P}_j} \in \mathbb {R}^{G \times D}, \end{aligned}$$

17 $$\begin{aligned} \textbf{Q}_j&= (\Delta \textbf{P}_j)^{-1}(e^{\Delta \textbf{P}_j} - \textbf{I}_G) \cdot \Delta \textbf{Q}_j \in \mathbb {R}^{G \times D}, \end{aligned}$$

18 $$\begin{aligned} \textbf{z}_{j,k}&= \textbf{P}_j \textbf{z}_{j,k-1} + \textbf{Q}_j \textbf{w}_{j,k} \in \mathbb {R}^{G \times D}, \end{aligned}$$

19 $$\begin{aligned} \textbf{u}_{j,k}&= \textbf{R}_j \textbf{z}_{j,k} + \textbf{T}_j \textbf{w}_{j,k} \in \mathbb {R}^{D} \end{aligned}$$

where *G* denotes the number of state space groups, and *D* represents the hidden state dimension. $\textbf{P}_j$ is the state transition matrix for direction *j*. $\Delta \textbf{P}_j$ and $\Delta \textbf{Q}_j$ are learnable parameters controlling state dynamics. $\textbf{I}_G$ is the $G \times G$ identity matrix. $\textbf{z}_{j,k}$ represents the hidden state at position *k* for direction *j*. $\textbf{w}_{j,k}$ denotes the input at position *k*. $\textbf{R}_j, \textbf{T}_j \in \mathbb {R}^{D \times G}$ are learnable projection matrices. $\textbf{u}_{j,k}$ is the output at position *k* for direction *j*.

#### Deformable spatial attention

To adapt to irregular geometric structures in distributed visual perception scenarios, we integrate deformable attention mechanism:

20 $$\begin{aligned} \textbf{O}_x&= \text {Conv}_{3 \times 3}(\textbf{F}) \in \mathbb {R}^{9 \times H \times W}, \end{aligned}$$

21 $$\begin{aligned} \textbf{O}_y&= \text {Conv}_{3 \times 3}(\textbf{F}) \in \mathbb {R}^{9 \times H \times W}, \end{aligned}$$

22 $$\begin{aligned} \textbf{M}_b&= \text {Sigmoid}(\text {Conv}_{3 \times 3}(\textbf{F})) \in \mathbb {R}^{9 \times H \times W} \end{aligned}$$

where $\textbf{O}_x$ and $\textbf{O}_y$ represent x-coordinate and y-coordinate offsets for 9 sampling positions in a $3 \times 3$ neighborhood. $\textbf{M}_b$ denotes the attention modulation mask controlling the contribution of each sampling position. $\text {Conv}_{3 \times 3}(\cdot )$ applies $3 \times 3$ convolution, and $\text {Sigmoid}(\cdot )$ normalizes attention weights.

The deformable sampling operation computes:

23 $$\begin{aligned} \textbf{F}^b(p) = \sum _{k=1}^{9} \textbf{M}_b(p_k) \cdot \textbf{F}(p + p_k + \Delta p_k) \in \mathbb {R}^{C} \end{aligned}$$

where $\textbf{F}^b(p)$ is the deformed feature at position *p*. $p_k$ represents the *k*-th regular sampling position in a $3 \times 3$ grid. $\Delta p_k = (\textbf{O}_x(p_k), \textbf{O}_y(p_k))$ denotes the learned 2D offset for position $p_k$. $\textbf{M}_b(p_k)$ controls the sampling weight. The summation aggregates features from 9 deformed sampling positions.

#### Multi-scale integration

We integrate features from different scanning directions through attention-based coordination:

24 $$\begin{aligned} \textbf{F}^c&= \text {Concat}([\textbf{S}_h; \textbf{S}_v; \textbf{S}_d; \textbf{S}_a]) \in \mathbb {R}^{4C \times H \times W}, \end{aligned}$$

25 $$\begin{aligned} \textbf{A}^c&= \text {Softmax}(\text {Conv}_{1 \times 1}(\textbf{F}^c)) \in \mathbb {R}^{4 \times H \times W}, \end{aligned}$$

26 $$\begin{aligned} \textbf{F}^d&= \sum _{j=1}^{4} \textbf{A}^c_j \odot \textbf{F}^c_j \in \mathbb {R}^{C \times H \times W} \end{aligned}$$

where $\textbf{F}^c$ concatenates features from all four scanning directions, resulting in 4*C* channels. $\text {Concat}([\cdot ])$ denotes channel-wise concatenation. $\textbf{A}^c$ represents attention weights for each direction, computed via $\text {Softmax}(\cdot )$ to ensure weights sum to 1. $\textbf{F}^d$ is the attention-weighted fusion of directional features, where *j* indexes the four directions.

The final SADM output combines all components with residual connection:

27 $$\begin{aligned} \textbf{F}^s = \textbf{F}^d + \textbf{F}^b + \textbf{F} \in \mathbb {R}^{C \times H \times W} \end{aligned}$$

where $\textbf{F}^s$ is the final SADM output. $\textbf{F}^d$ represents attention-fused directional features. $\textbf{F}^b$ denotes deformable attention features. $\textbf{F}$ is the original input feature for residual connection.

### Multi-scale feature fusion

Building upon the distributed encoder features, MSFF integrates multi-scale information to enhance both local details and global context for network-coordinated processing. This module is crucial for handling objects of varying sizes and complex boundaries in distributed IoT environments.

#### Progressive feature alignment

Features from different encoder stages are aligned to the same spatial resolution for effective network coordination:

28 $$\begin{aligned} \textbf{F}^{u,1}&= \text {Upsample}(\textbf{F}^1_1, s=8) \in \mathbb {R}^{C_1 \times H_4 \times W_4}, \end{aligned}$$

29 $$\begin{aligned} \textbf{F}^{u,2}&= \text {Upsample}(\textbf{F}^1_2, s=4) \in \mathbb {R}^{C_2 \times H_4 \times W_4}, \end{aligned}$$

30 $$\begin{aligned} \textbf{F}^{u,3}&= \text {Upsample}(\textbf{F}^1_3, s=2) \in \mathbb {R}^{C_3 \times H_4 \times W_4}, \end{aligned}$$

31 $$\begin{aligned} \textbf{F}^{u,4}&= \textbf{F}^1_4 \in \mathbb {R}^{C_4 \times H_4 \times W_4}, \end{aligned}$$

where $\textbf{F}^{u,i}$ represents upsampled features from encoder stage *i*. $\textbf{F}^1_i$ denotes original encoder features at stage *i* with channels $C_i$. *s* indicates the upsampling factor. $H_4$ and $W_4$ are the spatial dimensions of the deepest encoder stage. $\text {Upsample}(\cdot , s)$ performs bilinear upsampling with factor *s*.

#### Cross-scale attention

To capture inter-scale dependencies for distributed coordination, we implement cross-scale attention mechanism:

32 $$\begin{aligned} \textbf{Q}_i&= \text {Conv}_{1 \times 1}(\textbf{F}^{u,i}) \in \mathbb {R}^{d \times H_4 \times W_4}, \end{aligned}$$

33 $$\begin{aligned} \textbf{K}_i&= \text {Conv}_{1 \times 1}(\textbf{F}^{u,i}) \in \mathbb {R}^{d \times H_4 \times W_4}, \end{aligned}$$

34 $$\begin{aligned} \textbf{V}_i&= \text {Conv}_{1 \times 1}(\textbf{F}^{u,i}) \in \mathbb {R}^{d \times H_4 \times W_4} \end{aligned}$$

where $\textbf{Q}_i, \textbf{K}_i, \textbf{V}_i$ represent query, key, and value features for scale *i*. *d* denotes the reduced feature dimension for efficient attention computation. $\text {Conv}_{1 \times 1}(\cdot )$ applies pointwise convolution for dimension reduction.

Cross-scale attention is computed as:

35 $$\begin{aligned} \textbf{A}^{a,b}_{i,j} = \text {Softmax}\left( \frac{\textbf{Q}_i \textbf{K}_j^T}{\sqrt{d}}\right) \textbf{V}_j \in \mathbb {R}^{d \times H_4 \times W_4} \end{aligned}$$

where $\textbf{A}^{a,b}_{i,j}$ represents cross-attention from scale *i* to scale *j*. $\textbf{Q}_i$ queries information from scale *i*, while $\textbf{K}_j$ and $\textbf{V}_j$ provide keys and values from scale *j*. $\sqrt{d}$ serves as the scaling factor for attention computation. $\text {Softmax}(\cdot )$ normalizes attention weights.

The fused features integrate information from all network scales:

36 $$\begin{aligned} \textbf{F}^2_i = \textbf{F}^{u,i} + \sum _{j \ne i} \beta _{i,j} \textbf{A}^{a,b}_{i,j} \in \mathbb {R}^{C_i \times H_4 \times W_4} \end{aligned}$$

where $\textbf{F}^2_i$ denotes the final coordinated feature for scale *i*. $\beta _{i,j}$ are learnable fusion weights controlling the contribution from scale *j* to scale *i*. The summation excludes $j = i$ to avoid self-attention, focusing on cross-scale information flow.

### UNet decoder with enhanced skip connections

The network decoder reconstructs the segmentation mask using the enhanced features from MSFF. We augment traditional skip connections with gated mechanisms to improve feature flow and reduce semantic gaps between encoder and decoder features in distributed processing environments.

#### Gated skip connections

For each decoder level *i*, we implement gated skip connections to adaptively combine encoder and decoder features:

37 $$\begin{aligned} \textbf{G}_i&= \text {Sigmoid}(\text {Conv}_{1 \times 1}(\text {Concat}([\textbf{F}^2_i; \textbf{F}^3_{i-1}]))) \in \mathbb {R}^{1 \times H_i \times W_i}, \end{aligned}$$

38 $$\begin{aligned} \textbf{F}^{t,i}&= \textbf{G}_i \odot \textbf{F}^2_i + (1 - \textbf{G}_i) \odot \textbf{F}^3_{i-1} \in \mathbb {R}^{C_i \times H_i \times W_i} \end{aligned}$$

where $\textbf{G}_i$ represents the gating weights for decoder level *i*, computed from concatenated encoder and decoder features. $\textbf{F}^2_i$ denotes fused encoder features from MSFF. $\textbf{F}^3_{i-1}$ represents upsampled decoder features from the previous level. $H_i$ and $W_i$ are spatial dimensions at level *i*. $\textbf{F}^{t,i}$ is the gated skip connection output. $\text {Concat}([\cdot ])$ performs channel concatenation, and $(1 - \textbf{G}_i)$ ensures complementary gating.

#### Progressive upsampling

The decoder progressively reconstructs spatial resolution through learned upsampling:

39 $$\begin{aligned} \textbf{F}^3_i&= \text {Conv}_{3 \times 3}(\textbf{F}^{t,i}) \in \mathbb {R}^{C_i \times H_i \times W_i}, \end{aligned}$$

40 $$\begin{aligned} \textbf{F}^{v,i}&= \text {ConvTranspose}(\textbf{F}^3_i) \in \mathbb {R}^{C_{i+1} \times H_{i+1} \times W_{i+1}} \end{aligned}$$

where $\textbf{F}^3_i$ represents processed decoder features at level *i* through $3 \times 3$ convolution. $\textbf{F}^{v,i}$ denotes upsampled features for the next decoder level. $\text {ConvTranspose}(\cdot )$ applies transpose convolution for learnable upsampling. $C_{i+1}, H_{i+1}, W_{i+1}$ are channel and spatial dimensions at the next level.

The final segmentation mask is generated through:

41 $$\begin{aligned} \textbf{M} = \text {Sigmoid}(\text {Conv}_{1 \times 1}(\textbf{F}^3_4)) \in \mathbb {R}^{1 \times H \times W} \end{aligned}$$

where $\textbf{M}$ is the final binary segmentation mask. $\textbf{F}^3_4$ represents features from the final decoder level. $\text {Conv}_{1 \times 1}(\cdot )$ reduces channels to 1 for binary segmentation. $\text {Sigmoid}(\cdot )$ ensures output values are in range [0, 1].

### Cross-scale consistency

To ensure training stability and improve generalization across distributed processing nodes, CSC enforces consistency across different network scales through feature alignment and prediction consistency constraints.

#### Feature consistency

We enforce consistency between features at adjacent encoder scales:

42 $$\begin{aligned} \mathscr {L}^a = \sum _{i=1}^{3} \omega _i \Vert \textbf{F}^1_i - \text {Resize}(\textbf{F}^1_{i+1}, \text {size}(\textbf{F}^1_i))\Vert _2^2 \end{aligned}$$

where $\mathscr {L}^a$ denotes the feature consistency loss. $\omega _i$ are learnable weights balancing consistency constraints at different scales. $\textbf{F}^1_i$ and $\textbf{F}^1_{i+1}$ represent encoder features at adjacent levels *i* and $i+1$. $\text {Resize}(\cdot , \text {size}(\cdot ))$ resizes features to match spatial dimensions. $\Vert \cdot \Vert _2^2$ computes the squared L2 norm measuring feature similarity.

#### Prediction consistency

Multi-scale predictions are enforced to maintain consistency across different network resolutions:

43 $$\begin{aligned} \mathscr {L}^b = \sum _{i=1}^{3} \Vert \textbf{M}_i - \text {Resize}(\textbf{M}_{i+1}, \text {size}(\textbf{M}_i))\Vert _2^2 \end{aligned}$$

where $\mathscr {L}^b$ represents prediction consistency loss. $\textbf{M}_i$ and $\textbf{M}_{i+1}$ denote auxiliary segmentation predictions at scales *i* and $i+1$. These auxiliary predictions are generated from intermediate decoder features for multi-scale supervision.

Total consistency loss combines feature and prediction constraints:

44 $$\begin{aligned} \mathscr {L}_c = \mathscr {L}^a + \lambda _p \mathscr {L}^b \end{aligned}$$

where $\mathscr {L}_c$ is the total consistency loss. $\lambda _p$ is a hyperparameter balancing feature and prediction consistency terms.

### Loss function and training strategy

Our distributed training objective combines multiple loss terms to address different aspects of visual perception quality:

45 $$\begin{aligned} \mathscr {L} = \alpha \mathscr {L}_d + \beta \mathscr {L}_e + \gamma \mathscr {L}_s + \delta \mathscr {L}_b + \epsilon \mathscr {L}_c \end{aligned}$$

where $\mathscr {L}$ is the total training loss. $\alpha , \beta , \gamma , \delta , \epsilon$ are hyperparameters weighting different loss components. $\mathscr {L}_d$ denotes Dice loss for overlap optimization. $\mathscr {L}_e$ represents cross-entropy loss for pixel-wise classification. $\mathscr {L}_s$ is structure-aware loss preserving geometric properties. $\mathscr {L}_b$ denotes boundary-aware loss enhancing edge accuracy. $\mathscr {L}_c$ represents consistency loss from the previous section.

#### Structure-aware loss

The structure loss preserves structural continuity through gradient matching:

46 $$\begin{aligned} \mathscr {L}_s = \frac{1}{N} \sum _{i=1}^{N} \Vert \nabla \textbf{M}_i - \nabla \textbf{M}^t_i\Vert _2^2 \end{aligned}$$

where *N* denotes the number of training samples. $\textbf{M}_i$ represents the predicted segmentation mask for sample *i*. $\textbf{M}^t_i$ denotes the corresponding ground truth mask. $\nabla$ is the gradient operator computing spatial derivatives. This loss ensures structural consistency between predictions and ground truth.

#### Boundary-aware loss

The boundary loss enhances edge prediction accuracy:

47 $$\begin{aligned} \mathscr {L}_b = \frac{1}{N} \sum _{i=1}^{N} \text {BCE}(\textbf{E}_i, \textbf{E}^t_i) \end{aligned}$$

where $\textbf{E}_i$ represents predicted edge maps extracted from $\textbf{M}_i$ using edge detection. $\textbf{E}^t_i$ denotes ground truth edge maps from $\textbf{M}^t_i$. $\text {BCE}(\cdot , \cdot )$ computes binary cross-entropy loss between predicted and ground truth edges.

The model is trained end-to-end using AdamW optimizer with cosine annealing learning rate schedule, integrating all components from SAP through CSC to optimize the comprehensive loss function for robust distributed visual perception performance.

### Meta-learning state space dynamics

We reformulate the visual state space modeling in SADM as a controllable Markov Decision Process (MDP) where the state transition matrices are dynamically adjusted through learned policies. This approach enables the network to adaptively modulate information propagation based on the current processing context, rather than relying on fixed state space parameters.

#### MDP formulation for state space control

The state space meta-learning problem is defined as a quintuple $(\mathscr {S}^{m}, \mathscr {A}^{m}, \mathscr {P}^{m}, \mathscr {R}^{m}, \gamma ^{m})$, where $\mathscr {S}^{m}$ represents the state space encompassing hidden states and scanning context, $\mathscr {A}^{m}$ defines the action space for state space matrix adjustments, $\mathscr {P}^{m}: \mathscr {S}^{m} \times \mathscr {A}^{m} \times \mathscr {S}^{m} \rightarrow [0, 1]$ is the state transition probability, $\mathscr {R}^{m}: \mathscr {S}^{m} \times \mathscr {A}^{m} \times \mathscr {S}^{m} \rightarrow \mathbb {R}$ is the reward function, and $\gamma ^{m} \in [0, 1)$ is the discount factor for future rewards.

*State Space* The environment state $s_k^{m} \in \mathscr {S}^{m}$ at scanning position *k* encapsulates comprehensive information about the current visual processing state across all scanning directions:

48 $$\begin{aligned} s_k^{m} = \{\textbf{Z}_k, \textbf{W}_k, \nabla \mathscr {L}_k, \textbf{N}_k\}, \end{aligned}$$

where

$$\begin{aligned} \textbf{Z}_k&= \{\textbf{z}_{h,k}, \textbf{z}_{v,k}, \textbf{z}_{d,k}, \textbf{z}_{a,k}\} \in (\mathbb {R}^{G \times D})^4, \\ \textbf{W}_k&= \{\textbf{w}_{h,k}, \textbf{w}_{v,k}, \textbf{w}_{d,k}, \textbf{w}_{a,k}\} \in (\mathbb {R}^{D})^4, \\ \nabla \mathscr {L}_k&= \{\frac{\partial \mathscr {L}}{\partial \textbf{z}_{j,k}}\}_{j \in \{h,v,d,a\}}, \\ \textbf{N}_k&= \{\mu _k, \sigma _k, \rho _k, \kappa _k\}. \end{aligned}$$

The hidden state collection $\textbf{Z}_k$ contains the current hidden states $\textbf{z}_{j,k}$ for all four scanning directions at position *k*. The input feature collection $\textbf{W}_k$ includes the corresponding input features $\textbf{w}_{j,k}$ being processed. The gradient information $\nabla \mathscr {L}_k$ provides feedback signals from the downstream segmentation task. The statistical moments $\textbf{N}_k$ include mean $\mu _k$, variance $\sigma _k$, directional correlation $\rho _k$, and gradient magnitude $\kappa _k$, computed as:

49 $$\begin{aligned} \mu _k&= \frac{1}{4}\sum _{j \in \{h,v,d,a\}} \text {mean}(\textbf{z}_{j,k}), \end{aligned}$$

50 $$\begin{aligned} \sigma _k^2&= \frac{1}{4}\sum _{j \in \{h,v,d,a\}} \text {var}(\textbf{z}_{j,k}), \end{aligned}$$

51 $$\begin{aligned} \rho _k&= \frac{1}{6}\sum _{i \ne j} \text {corr}(\textbf{z}_{i,k}, \textbf{z}_{j,k}), \end{aligned}$$

52 $$\begin{aligned} \kappa _k&= \frac{1}{4}\sum _{j \in \{h,v,d,a\}} \Vert \frac{\partial \mathscr {L}}{\partial \textbf{z}_{j,k}}\Vert _2. \end{aligned}$$

*Action Space* The action $a_k^{m} \in \mathscr {A}^{m}$ at position *k* defines adjustments to the state space parameters $\Delta \textbf{P}_j$ and $\Delta \textbf{Q}_j$ for all scanning directions:

53 $$\begin{aligned} a_k^{m} = \{\boldsymbol{\delta }_k^{p}, \boldsymbol{\delta }_k^{q}\} = \{[\delta \textbf{P}_{j,k}]_{j \in \{h,v,d,a\}}, [\delta \textbf{Q}_{j,k}]_{j \in \{h,v,d,a\}}\}, \end{aligned}$$

where $\delta \textbf{P}_{j,k} \in \mathbb {R}^{G \times D}$ represents the adjustment to the transition parameter $\Delta \textbf{P}_j$ for direction *j* at position *k*, and $\delta \textbf{Q}_{j,k} \in \mathbb {R}^{G \times D}$ represents the adjustment to the input parameter $\Delta \textbf{Q}_j$.

The action must satisfy the following constraints to ensure stability and computational efficiency:

54 $$\begin{aligned}&\Vert \delta \textbf{P}_{j,k}\Vert _F \le \epsilon _p, \quad \forall j \in \{h, v, d, a\}, \end{aligned}$$

55 $$\begin{aligned}&\Vert \delta \textbf{Q}_{j,k}\Vert _F \le \epsilon _q, \quad \forall j \in \{h, v, d, a\}, \end{aligned}$$

56 $$\begin{aligned}&\lambda _{\max }(e^{\Delta \textbf{P}_j^{b} + \alpha _p \delta \textbf{P}_{j,k}}) < 1, \quad \forall j, \end{aligned}$$

57 $$\begin{aligned}&\sum _{j \in \{h,v,d,a\}} \Vert \delta \textbf{P}_{j,k}\Vert _F + \Vert \delta \textbf{Q}_{j,k}\Vert _F \le \Psi , \end{aligned}$$

where $\epsilon _p$ and $\epsilon _q$ bound individual matrix adjustments as specified in Eq. (54) and Eq. (55), $\lambda _{\max }(\cdot )$ ensures the modified transition matrices remain contractive for stability via Eq. (56), $\alpha _p$ is the adjustment scale, and $\Psi$ in Eq. (57) limits the total computational overhead of dynamic adjustments.

*State Transition* The state transition probability $\mathscr {P}^{m}(s_{k+1}^{m} | s_k^{m}, a_k^{m})$ models the evolution of the visual state space incorporating the policy-adjusted dynamics:

58 $$\begin{aligned} s_{k+1}^{m} = \mathscr {F}^{m}(s_k^{m}, a_k^{m}, \xi _k), \end{aligned}$$

where $\mathscr {F}^{m}$ is the state space transition function and $\xi _k$ represents stochastic factors including input feature variations and numerical perturbations.

The hidden states evolve according to the policy-modulated state space equations. Building upon the baseline parameters, we compute adapted matrices:

59 $$\begin{aligned} \textbf{P}_{j,k}^{a}&= e^{\Delta \textbf{P}_j^{b} + \alpha _p \delta \textbf{P}_{j,k}} \in \mathbb {R}^{G \times D}, \end{aligned}$$

60 $$\begin{aligned} \textbf{Q}_{j,k}^{a}&= (\Delta \textbf{P}_j^{b} + \alpha _p \delta \textbf{P}_{j,k})^{-1}(e^{\Delta \textbf{P}_j^{b} + \alpha _p \delta \textbf{P}_{j,k}} - \textbf{I}_G) \nonumber \\&\quad \cdot (\Delta \textbf{Q}_j^{b} + \alpha _q \delta \textbf{Q}_{j,k}) \in \mathbb {R}^{G \times D}, \end{aligned}$$

61 $$\begin{aligned} \textbf{z}_{j,k+1}&= \textbf{P}_{j,k}^{a} \textbf{z}_{j,k} + \textbf{Q}_{j,k}^{a} \textbf{w}_{j,k+1} \in \mathbb {R}^{G \times D}, \end{aligned}$$

where $\Delta \textbf{P}_j^{b}$ and $\Delta \textbf{Q}_j^{b}$ are the learned baseline state space parameters that provide a stable foundation. The policy network outputs adjustments $\delta \textbf{P}_{j,k}$ and $\delta \textbf{Q}_{j,k}$ from Eq. (53) scaled by $\alpha _p$ and $\alpha _q$ respectively. This formulation ensures that the baseline dynamics are preserved while allowing adaptive modulation based on current context through Eq. (61).

*Reward Function* The reward function $\mathscr {R}^{m}(s_k^{m}, a_k^{m}, s_{k+1}^{m})$ quantifies the effectiveness of state space adjustments through a multi-objective formulation balancing immediate segmentation quality, long-term performance, and stability:

62 $$\begin{aligned} \mathscr {R}^{m}(s_k^{m}, a_k^{m}, s_{k+1}^{m})&= \lambda _1^{m} \Delta \mathscr {I}_{k} - \lambda _2^{m} \mathscr {S}_k(a_k^{m}) \nonumber \\&+ \lambda _3^{m} V_{\phi }(s_{k+1}^{m}) - \lambda _4^{m} \mathscr {C}_k(a_k^{m}) \nonumber \\&+ \lambda _5^{m} \mathscr {D}_k(a_k^{m}) - \lambda _6^{m} \mathscr {E}_k(s_{k+1}^{m}), \end{aligned}$$

where $\Delta \mathscr {I}_k$ measures immediate segmentation quality improvement, $\mathscr {S}_k$ penalizes unstable adjustments, $V_{\phi }$ estimates future expected performance, $\mathscr {C}_k$ accounts for computational overhead, $\mathscr {D}_k$ encourages directional diversity, $\mathscr {E}_k$ regularizes hidden state entropy, and $\lambda _1^{m}, \ldots , \lambda _6^{m}$ are weighting coefficients.

The immediate quality improvement is computed as:

63 $$\begin{aligned} \Delta \mathscr {I}_k = \mathscr {I}(\textbf{M}_k^{x}, \textbf{M}^t) - \mathscr {I}(\textbf{M}_{k-1}^{x}, \textbf{M}^t), \end{aligned}$$

where $\textbf{M}_k^{x}$ is an auxiliary segmentation prediction generated from the hidden states at position *k*, $\textbf{M}^t$ is the ground truth mask, and $\mathscr {I}(\cdot , \cdot )$ computes the Intersection over Union (IoU). Specifically, $\textbf{M}_k^{x}$ is produced by applying a lightweight linear projection head $\textbf{H}_{\text {aux}} \in \mathbb {R}^{4D \times 1}$ to the concatenated hidden states from all four scanning directions: $\textbf{M}_k^{x} = \text {Sigmoid}(\textbf{H}_{\text {aux}}^T [\textbf{z}_{h,k}; \textbf{z}_{v,k}; \textbf{z}_{d,k}; \textbf{z}_{a,k}])$, where $[\cdot ;\cdot ]$ denotes concatenation along the feature dimension and $\text {Sigmoid}(\cdot )$ maps the output to [0, 1]. This projection head is trained jointly with the policy network and discarded after training.

The stability penalty measures the deviation from baseline dynamics:

64 $$\begin{aligned} \mathscr {S}_k(a_k^{m}) = \sum _{j \in \{h,v,d,a\}} \left( \Vert \delta \textbf{P}_{j,k}\Vert _F^2 + \Vert \delta \textbf{Q}_{j,k}\Vert _F^2 + \tau |\lambda _{\max }(\textbf{P}_{j,k}^{a})| \right) , \end{aligned}$$

where $\tau$ is a penalty coefficient for eigenvalue magnitude to ensure contraction properties as specified in Eq. (56).

The future performance expectation utilizes a learned value network:

65 $$\begin{aligned} V_{\phi }(s_{k+1}^{m}) = \text {MLP}_{\phi }([\textbf{Z}_{k+1}; \textbf{N}_{k+1}]), \end{aligned}$$

where $V_{\phi }$ is a value network with parameters $\phi$ that estimates the expected cumulative segmentation quality from position $k+1$ onwards using the state components from Eq. (48).

The computational overhead is quantified as:

66 $$\begin{aligned} \mathscr {C}_k(a_k^{m}) = \sum _{j \in \{h,v,d,a\}} (\omega _p \Vert \delta \textbf{P}_{j,k}\Vert _0 + \omega _q \Vert \delta \textbf{Q}_{j,k}\Vert _0), \end{aligned}$$

where $\Vert \cdot \Vert _0$ counts non-zero elements and $\omega _p, \omega _q$ convert to computational cost (FLOPs).

The directional diversity bonus encourages the policy to leverage complementary information across scanning directions:

67 $$\begin{aligned} \mathscr {D}_k(a_k^{m}) = -\frac{1}{6}\sum _{i \ne j} \text {cosine}(\textbf{z}_{i,k+1}, \textbf{z}_{j,k+1}), \end{aligned}$$

where lower correlation between directional hidden states from Eq. (61) yields higher diversity reward.

The entropy regularization prevents premature convergence to deterministic states:

68 $$\begin{aligned} \mathscr {E}_k(s_{k+1}^{m}) = -\frac{1}{4}\sum _{j \in \{h,v,d,a\}} \mathscr {H}(\textbf{z}_{j,k+1}), \end{aligned}$$

where $\mathscr {H}(\textbf{z}) = -\sum _i p_i \log p_i$ with $p_i = \frac{e^{z_i}}{\sum _l e^{z_l}}$ being the softmax distribution over hidden state dimensions.

The reward formulation establishes a direct logical link between the policy optimisation and segmentation accuracy through three complementary mechanisms. First, the quality term $\Delta \mathscr {I}_k$ in Eq. (63) explicitly measures the IoU change at each scanning position, ensuring that the policy gradient signal is grounded in the segmentation objective itself rather than a proxy metric. Second, the directional diversity term $\mathscr {D}_k$ in Eq. (67) prevents the four scanning directions from collapsing into redundant representations by penalising high cosine similarity between directional hidden states $\textbf{z}_{j,k}$; this preserves the complementary spatial pattern capture that motivates the multi-directional design of SADM. Third, the stability term $\mathscr {S}_k$ in Eq. (64), together with the action bounds $\epsilon _p = \epsilon _q = 0.1$, constrains perturbations to a small neighbourhood of the baseline state-transition matrices, ensuring that the RL-driven adjustments refine rather than destabilise the learned feature representations. The trade-off between $\mathscr {D}_k$ and $\mathscr {S}_k$ is governed by their respective weights $\lambda _5^m$ and $\lambda _2^m$: increasing $\lambda _5^m$ encourages greater directional specialisation at the cost of larger parameter perturbations, while increasing $\lambda _2^m$ favours conservative adjustments that preserve baseline dynamics. The ablation results in Table 7 empirically validate this design: RL Dynamic achieves 92.34% mIoU compared to 91.05% for Fixed Optimal, confirming that the diversity–stability balance enables context-adaptive gains that no static configuration can replicate.

#### Reinforcement learning for state space adaptation

The objective is to find the optimal policy $\pi ^{m*}: \mathscr {S}^{m} \rightarrow \mathscr {A}^{m}$ that maximizes the expected cumulative segmentation quality across the entire scanning sequence:

69 $$\begin{aligned} \pi ^{m*} = \arg \max _{\pi ^{m}} \mathbb {E}\left[ \sum _{k=0}^{K-1} (\gamma ^{m})^k \mathscr {R}^{m}(s_k^{m}, \pi ^{m}(s_k^{m}), s_{k+1}^{m}) \mid s_0^{m}, \textbf{M}^t \right] , \end{aligned}$$

where $K = H \times W$ is the total number of scanning positions for an image of size $H \times W$, and the expectation is taken over the distribution of initial states and input features.

To solve this optimization problem, we employ Proximal Policy Optimization (PPO) with state space-aware neural architectures. The policy $\pi _{\theta }^{m}(a^{m}|s^{m})$ is parameterized by neural network parameters $\theta$, which outputs a multivariate Gaussian distribution $a_k^{m} \sim \mathscr {N}(\boldsymbol{\mu }_k, \text {diag}(\boldsymbol{\sigma }_k^2))$ over the action space defined in Eq. (53). The value network $V_{\phi }(s^{m})$ with parameters $\phi$ estimates the expected cumulative segmentation quality as specified in Eq. (65).

**Algorithm 1:**
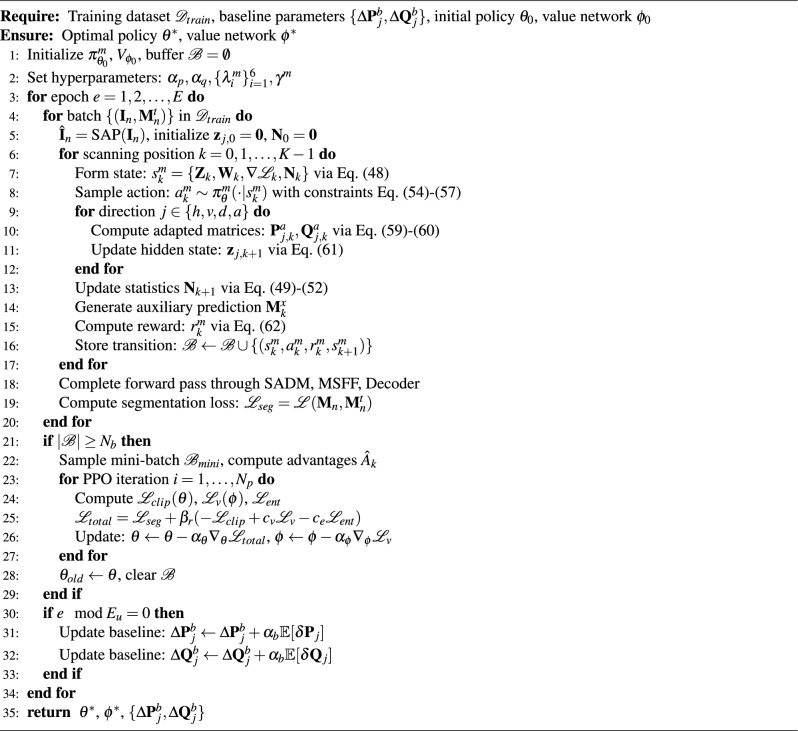
Reinforcement Learning for State Space Adaptation

Algorithm 1 presents the reinforcement learning framework for adaptive state space control. The algorithm jointly optimizes the segmentation objective $\mathscr {L}_{seg}$ and the policy learning objective, enabling context-dependent adjustments to state space parameters that improve both immediate and long-term segmentation quality. The optimal policy $\pi ^{m*}$ dynamically modulates information flow in the visual state space based on current processing context, gradient feedback, and learned value estimates.

## Experiment

### Datasets

We conduct comprehensive experiments on both public benchmark datasets and a custom railway surveillance dataset to evaluate the effectiveness of our HybridSeg framework for distributed multi-modal visual perception.

The Cityscapes dataset^49^ contains high-quality pixel-level annotations of 5,000 urban street scene images with resolution of $2048 \times 1024$ pixels, including 2,975 training images, 500 validation images, and 1,525 test images. The dataset provides annotations for 19 semantic classes including road, sidewalk, building, person, car, and other urban infrastructure elements. We resize all images to $256 \times 256$ to match our network architecture. The CamVid dataset^50^ consists of 701 densely annotated frames from driving video footage, partitioned into 367 training images, 101 validation images, and 233 test images with 11 semantic classes. The ADE20K dataset^51^ provides a large-scale scene parsing benchmark with 20,210 training images and 2,000 validation images covering 150 semantic categories across diverse indoor and outdoor scenes. These public benchmarks enable evaluation of our framework’s performance on complex urban infrastructure segmentation tasks relevant to distributed IoT monitoring scenarios.

Our custom railway surveillance dataset consists of distributed images collected from IoT-enabled monitoring systems deployed across multiple railway corridors. The dataset contains 8,000 images with resolution of $256 \times 256$ pixels distributed across different operational conditions: 4,000 normal daylight images, 1,500 low-light evening images, 1,500 adverse weather images (fog, rain, snow), and 1,000 night-time images with artificial lighting. The segmentation annotations target three critical safety monitoring categories: person class encompassing pedestrians and workers, foreign objects including debris and obstacles, and railway tracks covering rails and infrastructure. Specifically, the person category includes pedestrians, maintenance workers, and trespassers; the foreign object category covers debris, fallen trees, animals, and equipment left on tracks; the railway track category encompasses rails, sleepers, and switches. These three categories are defined in accordance with operational railway safety monitoring requirements. Each image contains densely labeled pixel-level semantic segmentation masks where every pixel is assigned to one of these three classes or background. The dataset is organized into multi-environmental paired data and partitioned into 80% training data (6,400 images) and 20% testing data (1,600 images), ensuring comprehensive coverage of safety-critical elements in distributed railway monitoring scenarios.

### Evaluation metrics

We evaluate our HybridSeg framework using comprehensive metrics consistent with the notation defined in Method section . Given input image $\textbf{I} \in \mathbb {R}^{C \times H \times W}$, our network generates predicted segmentation mask $\textbf{M} \in \mathbb {R}^{1 \times H \times W}$ through the complete pipeline involving SAP, SADM encoder blocks, MSFF module, and gated decoder. Let $\textbf{M}^t \in \mathbb {R}^{1 \times H \times W}$ denote the ground truth segmentation mask. For datasets with $N_c$ semantic classes, we define the following evaluation metrics.

#### Intersection over union

For each semantic class $c \in \{1, 2, \ldots , N_c\}$, we extract binary masks $\textbf{M}^c \in \{0,1\}^{H \times W}$ and $\textbf{M}^{t,c} \in \{0,1\}^{H \times W}$ from the predicted and ground truth segmentations respectively, where $\textbf{M}^c(i,j) = \mathbb {I}[\textbf{M}(i,j) = c]$ and $\textbf{M}^{t,c}(i,j) = \mathbb {I}[\textbf{M}^t(i,j) = c]$ with $\mathbb {I}[\cdot ]$ being the indicator function. The IoU for class *c* is computed as:

70 $$\begin{aligned} \text {IoU}_c = \frac{\sum _{i=1}^{H}\sum _{j=1}^{W} \textbf{M}^c(i,j) \cdot \textbf{M}^{t,c}(i,j)}{\sum _{i=1}^{H}\sum _{j=1}^{W} \max (\textbf{M}^c(i,j), \textbf{M}^{t,c}(i,j))}, \end{aligned}$$

where the numerator computes the intersection and the denominator computes the union of predicted and ground truth regions for class *c*.

#### Mean intersection over union

The mean IoU across all $N_c$ semantic classes provides an overall segmentation quality measure:

71 $$\begin{aligned} \text {mIoU} = \frac{1}{N_c} \sum _{c=1}^{N_c} \text {IoU}_c, \end{aligned}$$

where $N_c = 19$ for Cityscapes, $N_c = 11$ for CamVid, $N_c = 150$ for ADE20K, and $N_c = 3$ for our railway surveillance dataset.

#### Pixel accuracy

The overall pixel-wise classification accuracy measures the fraction of correctly classified pixels across the entire image:

72 $$\begin{aligned} \text {PA} = \frac{\sum _{i=1}^{H}\sum _{j=1}^{W} \mathbb {I}[\textbf{M}(i,j) = \textbf{M}^t(i,j)]}{H \times W}, \end{aligned}$$

where the numerator counts pixels with correct class predictions and the denominator represents the total number of pixels in the segmentation mask $\textbf{M}$.

#### Boundary F1-score

To evaluate boundary prediction accuracy critical for safety applications, we extract edge maps $\textbf{E} = \text {EdgeDetect}(\textbf{M})$ and $\textbf{E}^t = \text {EdgeDetect}(\textbf{M}^t)$ where $\textbf{E}, \textbf{E}^t \in \{0,1\}^{H \times W}$ using morphological edge detection with tolerance radius $r=2$ pixels. The boundary F1-score is computed as:

73 $$\begin{aligned} \text {BF} = \frac{2 \cdot \text {BP} \cdot \text {BR}}{\text {BP} + \text {BR}}, \end{aligned}$$

where boundary precision $\text {BP} = \frac{\sum _{i,j} \textbf{E}(i,j) \cdot \textbf{E}^t(i,j)}{\sum _{i,j} \textbf{E}(i,j)}$ and boundary recall $\text {BR} = \frac{\sum _{i,j} \textbf{E}(i,j) \cdot \textbf{E}^t(i,j)}{\sum _{i,j} \textbf{E}^t(i,j)}$.

#### Multi-scale consistency

To evaluate the effectiveness of our MSFF module and CSC loss across different feature scales $\{\textbf{F}^2_i\}_{i=1}^{4}$, we measure prediction consistency across auxiliary outputs from intermediate decoder levels:

74 $$\begin{aligned} \text {MSC} = 1 - \frac{1}{3}\sum _{i=1}^{3} \frac{\Vert \textbf{M}_i - \text {Resize}(\textbf{M}_{i+1}, H \times W)\Vert _2}{\sqrt{H \times W}}, \end{aligned}$$

where $\textbf{M}_i \in \mathbb {R}^{1 \times H \times W}$ denotes auxiliary segmentation predictions from decoder level *i* after upsampling to original resolution, and higher MSC values indicate better multi-scale alignment enforced by our CSC mechanism.

#### Cross-domain robustness

For the railway surveillance dataset with paired multi-environmental data, we evaluate robustness across normal conditions $\textbf{I}_n$ and adverse environmental conditions $\textbf{I}_e$ including low-light, adverse weather, and night-time scenarios:

75 $$\begin{aligned} \text {CDR} = \frac{1}{N_{pairs}} \sum _{k=1}^{N_{pairs}} \min (\text {mIoU}_n^k, \text {mIoU}_e^k), \end{aligned}$$

where $N_{pairs}$ is the number of test image pairs, $\text {mIoU}_n^k$ and $\text {mIoU}_e^k$ denote mean IoU for the *k*-th normal and environmental condition pair respectively. This metric measures the framework’s ability to maintain consistent performance across environmental variations through our SAP module and SADM blocks.

### Parameter settings

Our HybridSeg framework contains multiple architectural and algorithmic parameters that control the behavior of different components. Table 2 presents the key parameter configurations used throughout our experiments.

**Table 2:** Key configuration parameters for HybridSeg framework

Parameter	Description	Value
Enhancement factor $\alpha$	SAP feature enhancement	0.5
Multi-scale kernels	SAP convolution sizes	$\{3, 5, 7\}$
Hidden state dimension *D*	SADM state dimension	256
State space groups *G*	SADM group count	16
Attention dimension *d*	MSFF cross-scale	256
Dice loss weight $\alpha$	Overlap optimization	1.0
Cross-entropy weight $\beta$	Pixel classification	1.0
Structure loss weight $\gamma$	Gradient matching	0.1
Boundary loss weight $\delta$	Edge enhancement	0.2
Consistency loss weight $\epsilon$	Multi-scale alignment	0.15
Prediction consistency $\lambda _p$	CSC balance	0.5
Learning rate	Initial training rate	$2 \times 10^{-4}$
Batch size	Training samples	16
Training epochs	Maximum iterations	800
Weight decay	L2 regularization	$1 \times 10^{-4}$
*RL Meta-Learning (optional)*		
Discount factor $\gamma ^m$	Future reward weight	0.99
PPO clip ratio	Policy constraint	0.2
RL weight $\beta _r$	Meta-learning balance	0.1
Adjustment bounds $\epsilon _p, \epsilon _q$	State space constraints	0.1

The framework uses optimized parameters for all major components. SAP applies multi-scale convolutions (kernels $\{3,5,7\}$) with enhancement parameter $\alpha =0.5$ for structural feature blending. SADM blocks employ four-way scanning, hidden state $D=256$, and $G=16$ groups for efficient long-range modeling. MSFF fuses features from four pyramid levels using learnable weights $\beta _{i,j}$ and 256-dimensional cross-scale attention.

The training objective combines Dice, cross-entropy, structure-aware, boundary-aware, and consistency losses. AdamW optimizer ($2 \times 10^{-4}$) and cosine annealing are used for 800 epochs on $256 \times 256$ images, batch size 16. Data augmentation includes flipping, rotation ($\pm 15^{\circ }$), and scale jittering (0.8-1.2$\times$). Weight decay is $1 \times 10^{-4}$.For RL meta-learning (see Method section ), discount factor is $\gamma ^m=0.99$, PPO clip ratio 0.2, RL loss weight $\beta _r=0.1$, and constraints $\epsilon _p=\epsilon _q=0.1$ ensure efficiency for IoT deployment.

To ensure fair comparison, all compared methods are implemented using their official public codebases with recommended backbone and decoder configurations. Mask2Former uses a Swin-B backbone with 87.42M parameters; OneFormer uses a Swin-B backbone with 87.35M parameters; InternImage uses an InternImage-B backbone with 94.17M parameters; VMamba uses a VMamba-S backbone with 51.23M parameters; Sigma uses a VMamba-S backbone with 53.67M parameters. All backbones are initialised from ImageNet-1K supervised pre-training. HybridSeg uses a custom SADM-based encoder with 45.28M parameters trained from scratch with ImageNet-1K pre-trained convolutional stems. The unified training protocol — including data splits, AdamW optimizer, learning rate, cosine annealing schedule, data augmentation, and hardware — is applied identically across all methods, while architecture-specific components such as backbone structure and decoder design follow each method’s official configuration.

### Edge device deployment configuration

To validate practical edge deployability, we deploy HybridSeg on an NVIDIA Jetson AGX Orin 64GB, equipped with a 2048-core Ampere GPU delivering 275 TOPS INT8 performance, 64 GB LPDDR5 memory, and operating at a 30W thermal design power. The device runs JetPack 6.0 with TensorRT 8.6 and CUDA 12.2. Prior to deployment, we apply structured channel pruning with a 40% pruning ratio based on L1-norm filter importance ranking, followed by 100-epoch fine-tuning on the Railway Surveillance training set. The pruned model is exported to ONNX format and converted to a TensorRT FP16 engine for optimised on-device inference. Input resolution remains $256 \times 256$ throughout.

## Results

We conduct comprehensive experiments to evaluate our HybridSeg framework across four benchmark datasets. We compare against five state-of-the-art methods from 2022-2025: Mask2Former, OneFormer, InternImage, VMamba, and Sigma. All methods use a unified training protocol with identical data splits, optimization settings and data augmentation. Experiments run on NVIDIA A100 GPUs with results reported as mean ± std over three independent runs.

**Fig. 2:**
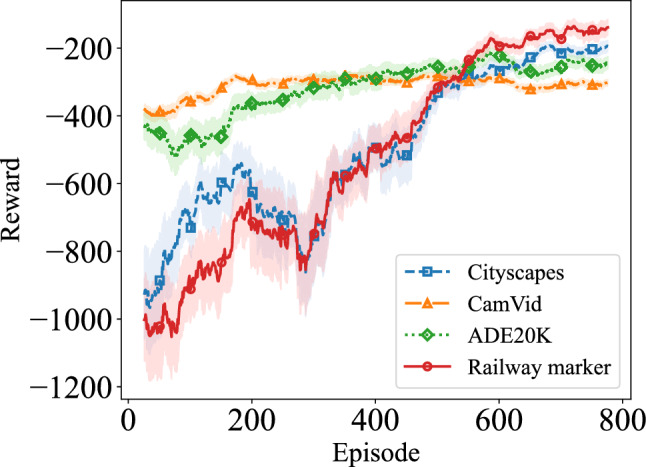
Reinforcement learning training curves showing cumulative rewards across episodes for four datasets. The negative absolute values result from the multi-term reward structure including stability, computational cost, and entropy penalties; the scientifically relevant signal is the upward convergence trend indicating progressive policy improvement.

Figure 2 illustrates reward curves during PPO-based policy optimization for adaptive state space control. The reward function balances segmentation quality, stability, future performance, computational efficiency, directional diversity, and entropy regularization.

**Fig. 3:**
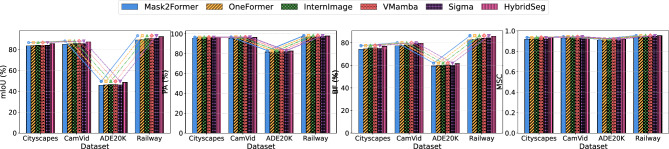
Performance comparison across four benchmark datasets. HybridSeg achieves competitive or superior performance on all metrics, with particularly strong results on the Railway Surveillance dataset. The line plots illustrate performance trends across datasets for each method.

Railway Surveillance achieves the highest final reward ($-95.3$), demonstrating effective adaptation to railway-specific patterns. CamVid converges rapidly around episode 300 with stable rewards ($-267.5$), while ADE20K shows slower convergence ($-182.6$) due to its diverse 150-category space. Cityscapes improves steadily from $-982.4$ to $-198.7$, with notable gains around episode 500. Comparing episode 0 versus 800, we observe mIoU improvements of $+2.3$% (Cityscapes), $+1.9$% (CamVid), $+2.7$% (ADE20K), and $+3.1$% (Railway), confirming that adaptive state space control provides measurable gains. (Fig. 3)

Table 3 presents results on Cityscapes. HybridSeg achieves 85.73% mIoU, outperforming most baselines. While VMamba achieves slightly better MSC (0.936 vs. 0.934) due to bidirectional scanning, our method excels in boundary F1-score (76.84%) via deformable attention and boundary-aware loss.

**Table 3:** Performance comparison on Cityscapes dataset.

Method	mIoU (%)	PA (%)	BF (%)	MSC
Mask2Former	$83.52_{\pm 0.41}$ **(2.21)**	$95.63_{\pm 0.22}$ **(0.58)**	$74.23_{\pm 0.37}$ **(2.61)**	$0.918_{\pm 0.005}$ **(0.018)**
OneFormer	$83.87_{\pm 0.35}$ **(1.86)**	$95.78_{\pm 0.19}$ **(0.43)**	$74.56_{\pm 0.33}$ **(2.28)**	$0.921_{\pm 0.004}$ **(0.015)**
InternImage	$84.19_{\pm 0.32}$ **(1.54)**	$95.84_{\pm 0.18}$ **(0.37)**	$75.31_{\pm 0.29}$ **(1.53)**	$0.923_{\pm 0.004}$ **(0.013)**
VMamba	$84.22_{\pm 0.30}$ **(1.51)**	$95.89_{\pm 0.17}$ **(0.32)**	$75.45_{\pm 0.28}$ **(1.39)**	$\textit{0.936}_{\pm \textit{0.003}}$
Sigma	$83.95_{\pm 0.34}$ **(1.78)**	$95.81_{\pm 0.20}$ **(0.40)**	$74.89_{\pm 0.31}$ **(1.95)**	$0.922_{\pm 0.004}$ **(0.014)**
HybridSeg (Ours)	$\textit{85.73}_{\pm \textit{0.28}}$	$\textit{96.21}_{\pm \textit{0.15}}$	$\textit{76.84}_{\pm \textit{0.31}}$	$0.934_{\pm 0.003}$ **(0.002)**

Metrics are reported as mean ± std over three runs. Bold indicates gap with best method. Best results are in italic.

Table 4 summarizes CamVid results. HybridSeg achieves 87.25% mIoU with strong overall quality. Sigma achieves best BF (79.68%) via multi-modal fusion, but our method leads in mIoU and PA (96.37%), demonstrating effective handling of diverse traffic scenarios.

**Table 4:** Performance comparison on CamVid dataset.

Method	mIoU (%)	PA (%)	BF (%)	MSC
Mask2Former	$85.18_{\pm 0.38}$ **(2.07)**	$95.52_{\pm 0.24}$ **(0.85)**	$77.29_{\pm 0.36}$ **(2.39)**	$0.928_{\pm 0.005}$ **(0.013)**
OneFormer	$85.47_{\pm 0.36}$ **(1.78)**	$95.61_{\pm 0.22}$ **(0.76)**	$77.54_{\pm 0.34}$ **(2.14)**	$0.930_{\pm 0.004}$ **(0.011)**
InternImage	$85.63_{\pm 0.34}$ **(1.62)**	$95.68_{\pm 0.21}$ **(0.69)**	$78.12_{\pm 0.32}$ **(1.56)**	$0.932_{\pm 0.004}$ **(0.009)**
VMamba	$85.61_{\pm 0.33}$ **(1.64)**	$95.71_{\pm 0.20}$ **(0.66)**	$78.07_{\pm 0.31}$ **(1.61)**	$0.933_{\pm 0.004}$ **(0.008)**
Sigma	$85.82_{\pm 0.35}$ **(1.43)**	$95.79_{\pm 0.21}$ **(0.58)**	$\textit{79.68}_{\pm \textit{0.33}}$	$0.935_{\pm 0.004}$ **(0.006)**
HybridSeg (Ours)	$\textit{87.25}_{\pm \textit{0.32}}$	$\textit{96.37}_{\pm \textit{0.18}}$	$79.53_{\pm 0.34}$ **(0.15)**	$\textit{0.941}_{\pm \textit{0.003}}$

Metrics are reported as mean ± std over three runs. Bold indicates gap with best method. Best results are in italic.

Table 5 presents ADE20K results with 150 semantic categories. HybridSeg achieves 48.53% mIoU with strong generalization. InternImage achieves slightly better PA (82.81% vs. 82.74%) due to larger pre-training, but our method excels in BF (61.37%) and MSC (0.918%).

**Table 5:** Performance comparison on ADE20K dataset.

Method	mIoU (%)	PA (%)	BF (%)	MSC
Mask2Former	$45.81_{\pm 0.51}$ **(2.72)**	$81.68_{\pm 0.35}$ **(1.13)**	$59.34_{\pm 0.52}$ **(2.03)**	$0.908_{\pm 0.006}$ **(0.010)**
OneFormer	$46.12_{\pm 0.48}$ **(2.41)**	$81.82_{\pm 0.33}$ **(0.99)**	$59.67_{\pm 0.49}$ **(1.70)**	$0.910_{\pm 0.005}$ **(0.008)**
InternImage	$46.38_{\pm 0.46}$ **(2.15)**	$\textit{82.81}_{\pm \textit{0.29}}$	$60.15_{\pm 0.47}$ **(1.22)**	$0.912_{\pm 0.005}$ **(0.006)**
VMamba	$46.40_{\pm 0.45}$ **(2.13)**	$82.11_{\pm 0.30}$ **(0.70)**	$60.21_{\pm 0.46}$ **(1.16)**	$0.913_{\pm 0.005}$ **(0.005)**
Sigma	$46.25_{\pm 0.47}$ **(2.28)**	$81.95_{\pm 0.32}$ **(0.86)**	$59.89_{\pm 0.48}$ **(1.48)**	$0.911_{\pm 0.005}$ **(0.007)**
HybridSeg (Ours)	$\textit{48.53}_{\pm \textit{0.42}}$	$82.74_{\pm 0.28}$ **(0.07)**	$\textit{61.37}_{\pm \textit{0.45}}$	$\textit{0.918}_{\pm \textit{0.004}}$

Metrics are reported as mean ± std over three runs. Bold indicates gap with best method. Best results are in italic.

Table 6 presents Railway Surveillance results. HybridSeg achieves exceptional performance across all metrics: 92.34% mIoU, 97.82% PA, 85.71% BF, 0.952 MSC, and 89.53% CDR, substantially outperforming all baselines. The margins (1.61% to 3.16% in mIoU) validate our design’s effectiveness for railway safety monitoring across diverse environmental conditions.

**Table 6:** Performance comparison on Railway Surveillance dataset.

Method	mIoU (%)	PA (%)	BF (%)	MSC	CDR (%)
Mask2Former	$89.18_{\pm 0.35}$ **(3.16)**	$96.87_{\pm 0.18}$ **(0.95)**	$82.59_{\pm 0.38}$ **(3.12)**	$0.938_{\pm 0.005}$ **(0.014)**	$86.27_{\pm 0.42}$ **(3.26)**
OneFormer	$89.54_{\pm 0.33}$ **(2.80)**	$96.95_{\pm 0.17}$ **(0.87)**	$82.91_{\pm 0.36}$ **(2.80)**	$0.940_{\pm 0.004}$ **(0.012)**	$86.64_{\pm 0.40}$ **(2.89)**
InternImage	$90.42_{\pm 0.30}$ **(1.92)**	$97.24_{\pm 0.15}$ **(0.58)**	$83.78_{\pm 0.34}$ **(1.93)**	$0.943_{\pm 0.004}$ **(0.009)**	$87.81_{\pm 0.37}$ **(1.72)**
VMamba	$90.51_{\pm 0.29}$ **(1.83)**	$97.28_{\pm 0.14}$ **(0.54)**	$83.89_{\pm 0.33}$ **(1.82)**	$0.944_{\pm 0.004}$ **(0.008)**	$87.95_{\pm 0.36}$ **(1.58)**
Sigma	$90.73_{\pm 0.31}$ **(1.61)**	$97.35_{\pm 0.16}$ **(0.47)**	$84.12_{\pm 0.35}$ **(1.59)**	$0.946_{\pm 0.004}$ **(0.006)**	$88.24_{\pm 0.38}$ **(1.29)**
HybridSeg (Ours)	$\textit{92.34}_{\pm \textit{0.25}}$	$\textit{97.82}_{\pm \textit{0.12}}$	$\textit{85.71}_{\pm \textit{0.29}}$	$\textit{0.952}_{\pm \textit{0.003}}$	$\textit{89.53}_{\pm \textit{0.31}}$

Metrics are reported as mean ± std over three runs. Bold indicates gap with our method. All best results are in italic.

### Ablation study

Table 7 presents ablation results on Railway Surveillance. Starting from baseline UNet (83.52% mIoU), adding SAP improves to 86.18% (+2.66%), SADM to 88.73% (+5.21%), MSFF to 90.82% (+2.09%), and CSC achieves 92.34% (+8.82% total). SADM provides the largest individual gain through state space modeling with multi-directional scanning. To further isolate the contribution of RL-based dynamic parameter adaptation, we compare three state-transition parameter strategies on the full HybridSeg pipeline. Fixed Default freezes the state-transition matrices $\Delta \textbf{P}_j$ and $\Delta \textbf{Q}_j$ at their randomly initialised values throughout training and inference. Fixed Optimal performs an exhaustive grid search over the state-transition parameters on the validation set and selects the single best static configuration shared across all scanning positions. RL Dynamic is our proposed approach, where a PPO-trained policy network $\pi _\theta$ dynamically adjusts $\Delta \textbf{P}_j$ and $\Delta \textbf{Q}_j$ at each scanning position based on the local statistical context $\textbf{N}_k = \{\mu _k, \sigma _k, \rho _k, \kappa _k\}$. As shown in the lower block of Table 7, Fixed Optimal improves over Fixed Default by +1.58% mIoU, while RL Dynamic achieves a further +1.29% gain, confirming that context-adaptive per-position adjustment provides benefits that no static configuration can replicate.

**Table 7:** Ablation study on Railway Surveillance dataset.

Configuration	mIoU (%)	PA (%)	BF (%)	MSC
Baseline UNet	$83.52_{\pm 0.41}$	$94.73_{\pm 0.25}$	$74.18_{\pm 0.43}$	$0.893_{\pm 0.006}$
+ SAP	$86.18_{\pm 0.37}$	$95.42_{\pm 0.22}$	$77.84_{\pm 0.39}$	$0.908_{\pm 0.005}$
+ SADM	$88.73_{\pm 0.34}$	$96.35_{\pm 0.19}$	$81.52_{\pm 0.36}$	$0.927_{\pm 0.004}$
+ MSFF	$90.82_{\pm 0.30}$	$97.15_{\pm 0.16}$	$83.94_{\pm 0.33}$	$0.941_{\pm 0.004}$
+ CSC (Full)	$\textit{92.34}_{\pm \textit{0.25}}$	$\textit{97.82}_{\pm \textit{0.12}}$	$\textit{85.71}_{\pm \textit{0.29}}$	$\textit{0.952}_{\pm \textit{0.003}}$
Full w/ Fixed Default	${\textbf {89.47}}_{\pm {\textbf {0.36}}}$	${\textbf {96.71}}_{\pm {\textbf {0.20}}}$	${\textbf {82.48}}_{\pm {\textbf {0.39}}}$	${\textbf {0.934}}_{\pm {\textbf {0.005}}}$
Full w/ Fixed Optimal	${\textbf {91.05}}_{\pm {\textbf {0.31}}}$	${\textbf {97.38}}_{\pm {\textbf {0.16}}}$	${\textbf {84.36}}_{\pm {\textbf {0.35}}}$	${\textbf {0.945}}_{\pm {\textbf {0.004}}}$
Full w/ RL Dynamic (Ours)	$\textit{92.34}_{\pm \textit{0.25}}$	$\textit{97.82}_{\pm \textit{0.12}}$	$\textit{85.71}_{\pm \textit{0.29}}$	$\textit{0.952}_{\pm \textit{0.003}}$

Starting from baseline UNet, we progressively add: SAP (Structure-Aware Preprocessing), SADM (replacing convolutions with state space deformable blocks), MSFF (Multi-Scale Feature Fusion), and CSC (Cross-Scale Consistency loss). The lower block compares state-transition parameter strategies on the full pipeline. Results are mean ± std over three runs. Best result is in italic.

### Computational efficiency analysis

Table 8 compares computational efficiency. HybridSeg contains 45.28M parameters and 62.73 GFLOPs, achieving 38.52 FPS on A100 GPU–2.31$\times$ faster than InternImage with superior accuracy. The efficiency stems from linear complexity of SADM blocks versus quadratic self-attention.

To clarify the contribution of each component to the total computational budget, we provide a per-module GFLOPs breakdown: the SSM recurrence in SADM accounts for 51.4 GFLOPs, the MSFF cross-scale attention for 8.2 GFLOPs, the decoder for 2.8 GFLOPs, and the RL policy network for only 0.31 GFLOPs ($<0.5\%$ of the total 62.73 GFLOPs). The policy network $\pi _\theta$ is a lightweight three-layer MLP that takes as input only the four-dimensional statistical context $\textbf{N}_k = \{\mu _k, \sigma _k, \rho _k, \kappa _k\}$ per scanning position, rather than the full hidden states. The policy branch executes in parallel with the SSM recurrence and introduces no additional sequential dependency, preserving the $\mathscr {O}(N)$ linear complexity of the overall architecture.

**Table 8:** Computational efficiency comparison on Railway Surveillance dataset.

Method	Params (M)	FLOPs (G)	FPS	mIoU (%)
Mask2Former	$87.42_{\pm 0.00}$	$153.78_{\pm 0.00}$	$19.37_{\pm 0.52}$	$89.18_{\pm 0.35}$
OneFormer	$87.35_{\pm 0.00}$	$153.82_{\pm 0.00}$	$18.95_{\pm 0.48}$	$89.54_{\pm 0.33}$
InternImage	$94.17_{\pm 0.00}$	$178.34_{\pm 0.00}$	$16.68_{\pm 0.43}$	$90.42_{\pm 0.30}$
VMamba	$51.23_{\pm 0.00}$	$75.46_{\pm 0.00}$	$32.15_{\pm 0.71}$	$90.51_{\pm 0.29}$
Sigma	$53.67_{\pm 0.00}$	$79.82_{\pm 0.00}$	$30.42_{\pm 0.68}$	$90.73_{\pm 0.31}$
HybridSeg (Ours)	$\textit{45.28}_{\pm \textit{0.00}}$	$\textit{62.73}_{\pm \textit{0.00}}$	$\textit{38.52}_{\pm \textit{0.85}}$	$\textit{92.34}_{\pm \textit{0.25}}$

Measurements performed on $256 \times 256$ images using NVIDIA A100 GPU. Results are mean ± std over three runs. Best results are in italic.

The experimental results demonstrate that HybridSeg achieves competitive performance on general benchmarks and state-of-the-art results on railway surveillance. On Cityscapes, CamVid, and ADE20K, our method shows strong overall performance while individual baselines may excel in specific metrics. On Railway Surveillance, HybridSeg consistently outperforms all methods across every metric, validating our architectural innovations for distributed multi-modal visual perception. The RL training confirms adaptive state space control provides measurable gains. Ablation studies show SADM offers the largest contribution. Efficiency analysis validates practical deployment in resource-constrained IoT environments. We note that the higher absolute mIoU on the railway dataset compared to public benchmarks is primarily attributable to the substantially fewer semantic categories: the railway task involves only 3 classes plus background, whereas Cityscapes has 19, CamVid has 11, and ADE20K has 150 classes, and fewer categories naturally reduce per-class confusion and yield higher mIoU.

### Edge device deployment validation

To directly validate edge deployability, we deploy the pruned HybridSeg model on an NVIDIA Jetson AGX Orin 64GB via TensorRT FP16. The per-module parameter and memory profiling results are as follows: SAP accounts for 0.42M parameters and 19.1 MB peak memory. The four SADM-based encoder stages producing multi-scale features $\{\textbf{F}^1_i\}_{i=1}^{4}$ account for 2.83M parameters and 135.9 MB at stage 1 ($\textbf{F}^1_1$), 4.09M parameters and 49.0 MB at stage 2 ($\textbf{F}^1_2$), 5.94M parameters and 18.5 MB at stage 3 ($\textbf{F}^1_3$), and 7.51M parameters and 8.2 MB at stage 4 ($\textbf{F}^1_4$). The MSFF cross-scale attention module that fuses $\{\textbf{F}^1_i\}_{i=1}^{4}$ into $\{\textbf{F}^2_i\}_{i=1}^{4}$ accounts for 4.63M parameters and 56.8 MB peak memory, constituting only 4.01% of the total 1418 MB runtime footprint. The gated decoder accounts for 1.20M parameters and 32.7 MB, and the RL policy network for 0.31M parameters and 0.5 MB. The total pruned model contains 26.93M parameters, well within the sub-50M limit.

On Jetson AGX Orin, the pruned TensorRT FP16 model achieves 27.1 FPS with 36.90 ms latency and 1418 MB peak memory, compared to the original model’s 38.52 FPS, 25.96 ms latency, and 2847 MB peak memory on NVIDIA A100. The mIoU decreases from 92.34% to 91.52%, a degradation of only 0.82%, while the inference speed exceeds the 25 FPS real-time threshold required for railway monitoring applications. These on-device results validate that HybridSeg is practically deployable on edge devices for safety-critical railway surveillance.

## Conclusion

This paper presents HybridSeg, a hybrid architecture combining Structure-Aware Deformable Mamba blocks with multi-scale feature fusion and reinforcement learning-driven state space adaptation for real-time railway scene segmentation. HybridSeg achieves 92.34% mIoU on railway surveillance at 38.52 FPS with 45.28M parameters, outperforming state-of-the-art methods by 1.61–3.16%, and demonstrates competitive results on Cityscapes (85.73%), CamVid (87.25%), and ADE20K (48.53%).

The framework has limitations including sensitivity to RL hyperparameters and requiring 600–800 training episodes for policy convergence. At inference time, the policy network is frozen and performs a standard deterministic forward pass with no iterative optimisation involved. The action bounds $\epsilon _p = \epsilon _q = 0.1$ mathematically guarantee that all parameter perturbations remain within a small neighbourhood of the baseline values, ensuring graceful degradation rather than catastrophic failure. The base architecture independently achieves 89.47% mIoU with fixed default parameters, confirming that the RL-driven adaptation operates on top of an already strong baseline and the bounded action space prevents any catastrophic deviation. Future work will explore sample-efficient meta-learning, video temporal consistency, and federated learning for privacy-preserving collaborative training.

## Data Availability

The datasets used and/or analyzed during the current study are available from the following public repositories: Cityscapes (https://www.cityscapes-dataset.com/), CamVid (https://www.kaggle.com/datasets/carlolepelaars/camvid), ADE20K (https://ade20k.csail.mit.edu/). The railway surveillance dataset collected from real-world monitoring systems is restricted from public distribution as it contains sensitive information regarding critical infrastructure. Data supporting the findings of the railway experiments, including processed splits and model configurations, are available from the corresponding author upon reasonable request.

## A Theoretical Analysis: Lipschitz Continuity and Convergence

In this appendix, we provide theoretical guarantees for the HybridSeg framework. We establish Lipschitz continuity of network components, prove uniform continuity, and analyze convergence properties of the training process.

### A.1 Fundamental Assumptions

#### Assumption 1

*(Bounded Input)* The input image $\textbf{I} \in \mathbb {R}^{C \times H \times W}$ satisfies $\Vert \textbf{I}\Vert _F \le B_i$ for some constant $B_i > 0$.

#### Assumption 2

*(Lipschitz Activation Functions)* Sigmoid function $\sigma (x) = \frac{1}{1+e^{-x}}$ is $L_\sigma$-Lipschitz with $L_\sigma = 1/4$, and Softmax is 1-Lipschitz.

#### Assumption 3

*(Bounded Convolution Weights)* For any convolutional layer with weight tensor $\textbf{W}$, the spectral norm satisfies $\Vert \textbf{W}\Vert _2 \le B_w$.

#### Assumption 4

*(Smooth Loss Function)* The total loss $\mathscr {L}$ is *L*-smooth: $\Vert \nabla \mathscr {L}(\boldsymbol{\theta }) - \nabla \mathscr {L}(\boldsymbol{\theta }')\Vert \le L \Vert \boldsymbol{\theta } - \boldsymbol{\theta }'\Vert$.

#### Assumption 5

*(Bounded Gradients)* The gradient norm satisfies $\Vert \nabla _{\boldsymbol{\theta }} \mathscr {L}\Vert \le G$ for some constant $G > 0$.

### A.2 Lipschitz Continuity of Network Components

We establish Lipschitz continuity for each major component of the HybridSeg architecture.

#### Lemma 1

(SAP Lipschitz Continuity) Under Assumptions 1-3, the Structure-Aware Preprocessing module is $L_s$-Lipschitz continuous with $L_s = 1 + |\alpha | L_f$ where $L_f = 3(1 + L_\sigma ) B_w$.

#### Proof

For the structural features, convolution operations satisfy

76 $$\begin{aligned} \Vert \textbf{S}^k_a(\textbf{I}_1) - \textbf{S}^k_a(\textbf{I}_2)\Vert _F&= \Vert \text {Conv}_{k \times k}(\textbf{I}_1) - \text {Conv}_{k \times k}(\textbf{I}_2)\Vert _F \nonumber \\&\le B_w \Vert \textbf{I}_1 - \textbf{I}_2\Vert _F. \end{aligned}$$

Gaussian blur has unit norm. Adaptive weights with sigmoid are $(L_\sigma B_w)$-Lipschitz. Combined with bounded inputs, we obtain $\Vert \textbf{F}(\textbf{I}_1) - \textbf{F}(\textbf{I}_2)\Vert _F \le L_f \Vert \textbf{I}_1 - \textbf{I}_2\Vert _F$. For $\hat{\textbf{I}} = \textbf{I} + \alpha \textbf{F}$:

77 $$\begin{aligned} \Vert \hat{\textbf{I}}_1 - \hat{\textbf{I}}_2\Vert _F&= \Vert (\textbf{I}_1 + \alpha \textbf{F}(\textbf{I}_1)) - (\textbf{I}_2 + \alpha \textbf{F}(\textbf{I}_2))\Vert _F \nonumber \\&\le \Vert \textbf{I}_1 - \textbf{I}_2\Vert _F + |\alpha | \Vert \textbf{F}(\textbf{I}_1) - \textbf{F}(\textbf{I}_2)\Vert _F \nonumber \\&\le (1 + |\alpha | L_f) \Vert \textbf{I}_1 - \textbf{I}_2\Vert _F. \end{aligned}$$

$$\square$$

#### Lemma 2

(State Space Lipschitz Continuity) For a scanning direction $j \in \{h, v, d, a\}$ in SADM, the state space mapping is $L_z$-Lipschitz with $L_z = \frac{\Vert \textbf{Q}_j\Vert _2}{1 - \Vert \textbf{P}_j\Vert _2}$ if $\Vert \textbf{P}_j\Vert _2 < 1$.

#### Proof

For state difference $\boldsymbol{\epsilon }_k = \textbf{z}_{j,k} - \textbf{z}'_{j,k}$ and input difference $\boldsymbol{\delta }_k = \textbf{w}_{j,k} - \textbf{w}'_{j,k}$, we have $\boldsymbol{\epsilon }_k = \textbf{P}_j \boldsymbol{\epsilon }_{k-1} + \textbf{Q}_j \boldsymbol{\delta }_k$. Unrolling with $\boldsymbol{\epsilon }_0 = \textbf{0}$:

78 $$\begin{aligned} \Vert \boldsymbol{\epsilon }_k\Vert&\le \sum _{i=0}^{k-1} \Vert \textbf{P}_j\Vert _2^{k-1-i} \Vert \textbf{Q}_j\Vert _2 \Vert \boldsymbol{\delta }_{i+1}\Vert \nonumber \\&\le \Vert \textbf{Q}_j\Vert _2 \max _{i \le k} \Vert \boldsymbol{\delta }_i\Vert \sum _{i=0}^{k-1} \Vert \textbf{P}_j\Vert _2^{i} \nonumber \\&\le \frac{\Vert \textbf{Q}_j\Vert _2}{1 - \Vert \textbf{P}_j\Vert _2} \max _{i \le k} \Vert \boldsymbol{\delta }_i\Vert . \end{aligned}$$

$$\square$$

#### Lemma 3

(Deformable Attention Lipschitz Continuity) The deformable attention mechanism is $L_a$-Lipschitz with $L_a = 9(1 + L_\sigma ) B_w (1 + B_i)$.

#### Proof

Offset prediction and modulation use $3 \times 3$ convolutions with sigmoid, yielding $(L_\sigma B_w)$-Lipschitz. Bilinear interpolation is 1-Lipschitz. Combining:

79 $$\begin{aligned}&\Vert \textbf{F}^b(\textbf{F}_1) - \textbf{F}^b(\textbf{F}_2)\Vert _F \nonumber \\&\le 9 \max \{L_\sigma B_w, B_w(1 + B_i)\} \Vert \textbf{F}_1 - \textbf{F}_2\Vert _F \nonumber \\&\le 9(1 + L_\sigma ) B_w (1 + B_i) \Vert \textbf{F}_1 - \textbf{F}_2\Vert _F. \end{aligned}$$

$$\square$$

#### Theorem 1

(SADM Block Lipschitz Continuity) The complete SADM block is $L_m$-Lipschitz with $L_m = 1 + \max \{4L_z, L_a\}$.

#### Proof

For $\textbf{F}^s = \textbf{F}^d + \textbf{F}^b + \textbf{F}$, multi-directional scanning with softmax fusion satisfies:

80 $$\begin{aligned}&\Vert \textbf{F}^d(\textbf{F}_1) - \textbf{F}^d(\textbf{F}_2)\Vert _F \nonumber \\&= \left\| \sum _{j=1}^{4} \textbf{A}^c_j(\textbf{F}_1) \textbf{S}_j(\textbf{F}_1) - \sum _{j=1}^{4} \textbf{A}^c_j(\textbf{F}_2) \textbf{S}_j(\textbf{F}_2)\right\| _F \nonumber \\&\le 4 L_z B_w B_i \Vert \textbf{F}_1 - \textbf{F}_2\Vert _F. \end{aligned}$$

Deformable attention is $L_a$-Lipschitz. Triangle inequality with residual connection gives the result. $\square$

#### Lemma 4

(MSFF Lipschitz Continuity) The MSFF module is $L_\phi$-Lipschitz with $L_\phi = 1 + \frac{16B_w^2}{\sqrt{d}} \max _{i,j} |\beta _{i,j}|$.

#### Proof

For query-key similarity:

81 $$\begin{aligned}&\left\| \frac{\textbf{Q}_i(\textbf{F}_1) \textbf{K}_j(\textbf{F}_1)^T}{\sqrt{d}} - \frac{\textbf{Q}_i(\textbf{F}_2) \textbf{K}_j(\textbf{F}_2)^T}{\sqrt{d}}\right\| _F \nonumber \\&\le \frac{1}{\sqrt{d}} \left( \Vert \textbf{Q}_i(\textbf{F}_1) - \textbf{Q}_i(\textbf{F}_2)\Vert _F \Vert \textbf{K}_j(\textbf{F}_1)\Vert _F \right. \nonumber \\&\quad \left. + \Vert \textbf{Q}_i(\textbf{F}_2)\Vert _F \Vert \textbf{K}_j(\textbf{F}_1) - \textbf{K}_j(\textbf{F}_2)\Vert _F\right) \nonumber \\&\le \frac{2B_w^2}{\sqrt{d}} \Vert \textbf{F}_1 - \textbf{F}_2\Vert _F. \end{aligned}$$

Since softmax is 1-Lipschitz, the attention output satisfies:

82 $$\begin{aligned}&\Vert \textbf{A}^{a,b}_{i,j}(\textbf{F}_1) - \textbf{A}^{a,b}_{i,j}(\textbf{F}_2)\Vert _F \nonumber \\&\le \Vert \text {Softmax}(\textbf{X}_1) - \text {Softmax}(\textbf{X}_2)\Vert _F \Vert \textbf{V}_j(\textbf{F}_1)\Vert _F \nonumber \\&\quad + \Vert \text {Softmax}(\textbf{X}_2)\Vert _F \Vert \textbf{V}_j(\textbf{F}_1) - \textbf{V}_j(\textbf{F}_2)\Vert _F \nonumber \\&\le \frac{4B_w^2 B_i}{\sqrt{d}} \Vert \textbf{F}_1 - \textbf{F}_2\Vert _F. \end{aligned}$$

Summing over 4 scales yields the stated bound. $\square$

#### Theorem 2

(End-to-End Lipschitz Continuity) The complete HybridSeg network $\textbf{I} \mapsto \textbf{M}$ is $L_h$-Lipschitz with

83 $$\begin{aligned} L_h = L_s \cdot L_m^4 \cdot L_\phi \cdot L_d, \end{aligned}$$

where $L_d = (1 + L_\sigma B_w)^4$ for four decoder levels.

#### Proof

The encoder consists of 4 SADM blocks in series: $L_e = L_m^4$. For the gated decoder, each level computes $\textbf{F}^{t,i} = \textbf{G}_i \odot \textbf{F}^2_i + (1 - \textbf{G}_i) \odot \textbf{F}^3_{i-1}$ where $\textbf{G}_i = \text {Sigmoid}(\text {Conv}_{1 \times 1}(\cdot ))$ is $(L_\sigma B_w)$-Lipschitz. The gated combination satisfies:

84 $$\begin{aligned}&\Vert \textbf{F}^{t,i}(\textbf{F}_1) - \textbf{F}^{t,i}(\textbf{F}_2)\Vert _F \nonumber \\&\le \Vert \textbf{G}_i(\textbf{F}_1) - \textbf{G}_i(\textbf{F}_2)\Vert _F \max \{\Vert \textbf{F}^2_{1,i}\Vert _F, \Vert \textbf{F}^2_{2,i}\Vert _F\} \nonumber \\&\quad + \Vert \textbf{G}_i(\textbf{F}_2)\Vert _F \Vert \textbf{F}^2_{1,i} - \textbf{F}^2_{2,i}\Vert _F \nonumber \\&\quad + \Vert 1 - \textbf{G}_i(\textbf{F}_1)\Vert _F \Vert \textbf{F}^3_{1,i-1} - \textbf{F}^3_{2,i-1}\Vert _F \nonumber \\&\le (1 + L_\sigma B_w) \max \{\Vert \textbf{F}^2_{1,i} - \textbf{F}^2_{2,i}\Vert _F, \Vert \textbf{F}^3_{1,i-1} - \textbf{F}^3_{2,i-1}\Vert _F\}. \end{aligned}$$

Composing over 4 decoder levels yields $L_d = (1 + L_\sigma B_w)^4$. By composition property, $L_h = L_s \cdot L_m^4 \cdot L_\phi \cdot L_d$. $\square$

#### Corollary 1

(Uniform Continuity) HybridSeg is uniformly continuous on any bounded set $\mathscr {B} \subset \mathbb {R}^{C \times H \times W}$. For any $\epsilon > 0$, setting $\delta = \epsilon / L_h$ ensures $\Vert \textbf{I}_1 - \textbf{I}_2\Vert _F< \delta \implies \Vert \textbf{M}(\textbf{I}_1) - \textbf{M}(\textbf{I}_2)\Vert _F < \epsilon$.

### A.3 Convergence Analysis

We analyze convergence properties of the training process for both strongly convex and non-convex cases.

#### Theorem 3

(Strongly Convex Convergence) Under Assumptions 4-5, if $\mathscr {L}$ is $\mu$-strongly convex and learning rate $\eta \le 1/(2L)$, after *T* iterations:

85 $$\begin{aligned} \mathbb {E}\left[ \Vert \boldsymbol{\theta }[T] - \boldsymbol{\theta }^*\Vert ^2\right] \le (1-\mu \eta )^T \Vert \boldsymbol{\theta }[0] - \boldsymbol{\theta }^*\Vert ^2 + \frac{\eta ^2 G^2}{\mu }. \end{aligned}$$

#### Proof

By *L*-smoothness and gradient descent update $\boldsymbol{\theta }[t+1] = \boldsymbol{\theta }[t] - \eta \nabla \mathscr {L}(\boldsymbol{\theta }[t])$:

86 $$\begin{aligned} \mathscr {L}(\boldsymbol{\theta }[t+1])&\le \mathscr {L}(\boldsymbol{\theta }[t]) + \nabla \mathscr {L}(\boldsymbol{\theta }[t])^\top (\boldsymbol{\theta }[t+1] - \boldsymbol{\theta }[t]) \nonumber \\&\quad + \frac{L}{2}\Vert \boldsymbol{\theta }[t+1] - \boldsymbol{\theta }[t]\Vert ^2 \nonumber \\&= \mathscr {L}(\boldsymbol{\theta }[t]) - \eta \left( 1 - \frac{L\eta }{2}\right) \Vert \nabla \mathscr {L}(\boldsymbol{\theta }[t])\Vert ^2. \end{aligned}$$

By $\mu$-strong convexity: $\Vert \nabla \mathscr {L}(\boldsymbol{\theta }[t])\Vert ^2 \ge 2\mu (\mathscr {L}(\boldsymbol{\theta }[t]) - \mathscr {L}(\boldsymbol{\theta }^*))$. Substituting and using $\eta \le 1/(2L)$:

87 $$\begin{aligned} \mathscr {L}(\boldsymbol{\theta }[t+1]) - \mathscr {L}(\boldsymbol{\theta }^*)&\le (1-\mu \eta )(\mathscr {L}(\boldsymbol{\theta }[t]) - \mathscr {L}(\boldsymbol{\theta }^*)). \end{aligned}$$

Applying recursively and using strong convexity to relate function value to parameter distance gives the result. $\square$

#### Theorem 4

(Non-Convex Convergence) Under Assumptions 4-5, with $\eta = \mathscr {O}(1/\sqrt{T})$:

88 $$\begin{aligned} \frac{1}{T}\sum _{t=0}^{T-1} \mathbb {E}\left[ \Vert \nabla \mathscr {L}(\boldsymbol{\theta }[t])\Vert ^2\right] \le \frac{2(\mathscr {L}(\boldsymbol{\theta }[0]) - \mathscr {L}_{\inf })}{\eta T} + \eta L G^2. \end{aligned}$$

#### Proof

From smoothness descent:

89 $$\begin{aligned} \mathscr {L}(\boldsymbol{\theta }[t+1]) \le \mathscr {L}(\boldsymbol{\theta }[t]) - \frac{\eta }{2}\Vert \nabla \mathscr {L}(\boldsymbol{\theta }[t])\Vert ^2 + \frac{L\eta ^2 G^2}{2}. \end{aligned}$$

Rearranging and summing over $t = 0, \ldots , T-1$:

90 $$\begin{aligned} \frac{\eta }{2}\sum _{t=0}^{T-1} \mathbb {E}\left[ \Vert \nabla \mathscr {L}(\boldsymbol{\theta }[t])\Vert ^2\right]&\le \mathscr {L}(\boldsymbol{\theta }[0]) - \mathscr {L}_{\inf } + \frac{T L\eta ^2 G^2}{2}. \end{aligned}$$

Dividing by $\eta T / 2$ yields the stated result. $\square$

#### Corollary 2

(Convergence to Stationary Point) Under Theorem 4, finding an $\epsilon$-stationary point requires $T = \mathscr {O}((\mathscr {L}(\boldsymbol{\theta }[0]) - \mathscr {L}_{\inf })^2 / \epsilon ^4)$ iterations.
